# Differentiating mechanism from outcome for ancestry-assortative mating in admixed human populations

**DOI:** 10.1101/2024.06.06.597727

**Published:** 2024-06-06

**Authors:** Dashiell J. Massey, Zachary A. Szpiech, Amy Goldberg

**Affiliations:** 1 Department of Evolutionary Anthropology, Duke University, USA 27708; 2 Department of Biology, Pennsylvania State University, USA 16801; 3 Institute for Computational and Data Sciences, Pennsylvania State University, USA 16801

## Abstract

Population genetic theory, and the empirical methods built upon it, often assume that individuals pair randomly for reproduction. However, natural populations frequently violate this assumption, which may potentially confound genome-wide association studies, selection scans, and demographic inference. Within several recently admixed human populations, empirical genetic studies have reported a correlation in global ancestry proportion between spouses, referred to as ancestry-assortative mating. Here, we use forward genomic simulations to link correlations in ancestry between mates to the underlying mechanistic mate-choice process. We consider the impacts of two types of mate-choice model, using either ancestry-based preferences or social groups as the basis for mate pairing. We find that multiple mate-choice models can produce the same correlations in ancestry proportion between spouses; however, we also highlight alternative analytic approaches and circumstances in which these models may be distinguished. With this work, we seek to highlight potential pitfalls when interpreting correlations in empirical data as evidence for a particular model of human mating practices, as well as to offer suggestions toward development of new best practices for analysis of human ancestry-assortative mating.

## Introduction

Non-random mating has long been appreciated as an important source of genetic structure in natural populations (Fisher 1918; Wright 1921; Wright 1950; Nagylaki 1978). Positive assortative mating (hereafter, assortative mating), wherein genotypic or phenotypic trait values are positively correlated between mates, has been empirically observed across animal species (Jiang et al. 2013). Theoretical and empirical studies have demonstrated the consequences of assortative mating for speciation and hybridization (Kondrashov 1983; Otto et al. 2008; Tung et al. 2012; Schumer et al. 2017; Kopp et al. 2018; Powell et al. 2021; Muralidhar et al. 2022; Natola et al. 2022; Smadja et al. 2022; Robinson et al. 2023), and for the distributions of traits within populations (Wright 1921; Norris et al. 2019; Kim et al. 2021; Border et al. 2022; Muralidhar et al. 2022; Horwitz et al. 2023).

In non-human primates that live in complex and structured social environments, sociodemographic factors have been demonstrated to influence mate pair formation (Keddy-Hector 1992; Klinkova et al. 2005; Setchell and Jean Wickings 2006; Van Belle et al. 2009; Tung et al. 2012; Fogel et al. 2021). As such, when it comes to humans, assortative mating has been the purview not only of biologists, but also social scientists (*e.g.*, (Buss and Barnes 1986; Mare 1991; Kalmijn 1998; Luo and Klohnen 2005; Blossfeld 2009; Torche 2010; Schwartz 2013; Greenwood et al. 2014; Henz and Mills 2017; Smieja and Stolarski 2018; Chiappori 2020; De La Mare and Lee 2023)). Positive correlations have been reported between human spouses for a diverse array of phenotypes, including morphometric measurements, health outcomes, personality traits, lifestyle factors, age, socioeconomic status, educational attainment, religious affiliation, and language (Nagoshi et al. 1990; Robinson et al. 2017; Fibla et al. 2022; Horwitz et al. 2023; Yamamoto et al. 2023). These associations are likely driven by multiple generative processes, including individual mate-choice preferences, phenotypic convergence over time facilitated by cohabitation, and social structures that restrict or promote particular pairings. These processes may act individually or in concert. For instance, sociological literature on assortative mating by educational attainment has revealed the influence of both a preference for mate similarity (Kalmijn 1998) and social barriers to marriage across socioeconomic class (Torche 2010), and that the strength of assortment by education over time is sensitive to the degree of temporal overlap between the end of schooling and average age at the time of marriage (Mare 1991), suggesting that social milieu is likely also an important factor in structuring mate pair outcomes.

Within the field of population genetics, relatively less attention has been paid to understanding the contributions of these generative processes to producing the observed correlations between mating pairs. Indeed, mechanism and outcome are often conflated or not clearly delineated when discussing assortative mating, which obscures these multiple mechanisms that may contribute to mate similarity. When studies do consider mechanism, they typically draw on foundational work in the sexual selection literature modeling the effects of female choice on male phenotype (Lande 1981; Kirkpatrick 1982; Seger 1985), in which the mechanism driving assortative mating is assumed to be mate choice. To extend this framework beyond sexual dimorphism, the mate-choice mechanism is modeled as a preference for mates that “match” an individual’s own phenotype (Kopp et al. 2018; Goldberg et al. 2020; Kim et al. 2021; Muralidhar et al. 2022). However, some studies have suggested temporal structure in the mating process (Xie et al. 2015; Woodman et al. 2023) or non-uniform ability to attract mates (Burley 1983) can generate phenotypic correlations between mates without an explicit preference for phenotypic similarity. In this paper, we will use the term “assortative mating” to refer specifically to an empirical observation of greater resemblance between mates than expected by chance, agnostic to mechanism.

Reports of assortative mating by ancestry in humans provide a particularly intriguing example for exploring mechanism because genetic ancestry is a complex quantitative “phenotype” that involves every locus in the genome and that can only be ascertained by sequencing. In recently admixed human populations, in which individuals derive ancestry from multiple source populations, a positive correlation in global ancestry proportion between spouses has been observed. This phenomenon, referred to as ancestry-assortative mating, has been reported in multiple Latino populations (Risch et al. 2009; Zou et al. 2015; Spear et al. 2020; Mas Sandoval et al. 2023), as well as in African-Americans (Zaitlen et al. 2017; Avadhanam and Williams 2022), Cabo Verdeans (Korunes et al. 2022), and ni-Vanuatu (Arauna et al. 2022). Ancestry-associated mating patterns, inferred from excess sharing of single-nucleotide variants, have also been reported in human populations with more distant histories of admixture, *e.g.*, non-Hispanic white spouse pairs in the United States (Sebro et al. 2010; Domingue et al. 2014; Sebro et al. 2017). Empirical studies commonly use such correlations in ancestry between spouses to suggest that ancestry shapes mate choice, although this interaction would necessarily be mediated by a proxy phenotype of some type, not by quantitative ancestry proportion. However, the relative contributions of individual preferences and broader social mechanisms to generating these correlations are unclear.

Accounting for assortative mating is critical for accurate statistical and population genetic analysis in admixed human populations: it has been shown that assortative mating confounds genome-wide association studies (Howe et al. 2021; Border et al. 2022; Veller and Coop 2024) and heritability estimation (Sebro and Risch 2012; Tenesa et al. 2016; Huang et al. 2023). In addition, ancestry-assortative mating specifically has been shown to produce estimates for the timing of admixture that are too recent (Zaitlen et al. 2017; Goldberg et al. 2020; Korunes et al. 2022). However, it remains challenging to establish a null expectation in the presence of assortative mating without understanding the underlying mechanism at play.

To date, statistical methods to correct for ancestry-assortative mating in empirical data have tended to generate null models by simulating data to match the observed empirical correlation in ancestry proportion between mating pairs, often without a specific mechanistic model (Zaitlen et al. 2017; Spear et al. 2020; Huang et al. 2023; Pfennig and Lachance 2023). This approach makes two important assumptions: first, that the correlation coefficient for a given population has remained constant over time; and second, that matching the correlation coefficient is sufficient to recapitulate the dynamics of global and local ancestry. In contrast, theoretical population genetic studies have examined the behavior of these same parameters using mechanistic models of biased mate choice (Goldberg et al. 2020; Kim et al. 2021; Muralidhar et al. 2022). This approach assumes that ancestry-based preference is the primary mechanism driving non-random mating. These studies typically do not directly consider whether the model used results in an observed correlation in ancestry between mates, which leaves open the question of how applicable their conclusions are to the context of empirical studies of human ancestry-assortative mating.

Here, we used forward-in-time simulations to probe the relationship between mate-choice mechanism and observed correlation in ancestry, comparing variants of two classes of mate-choice model. The first model class considers only individual mate-choice preferences for similarity in ancestry proportion, treating genetic ancestry as a continuous quantitative trait. The second class considers only social groups, assigning each individual to one of two groups and limiting cross-group mating events. (Importantly, group membership is “inherited” randomly from one parent and group identities are initially associated with source populations; see Models.) We found that ancestry-similarity and social group models can produce similar correlations in ancestry between mates, suggesting that either or both mechanisms could be relevant in interpreting empirical data. Furthermore, the correlation coefficient was not constant over time in any of our simulations, and not all variants of the ancestry-similarity class or all parameter values of the social group model maintained sufficient population variance to sustain correlations in ancestry over tens of generations. Our results highlight important caveats about the assumptions behind both existing approaches and suggest additional analyses of empirical data that may help reveal the underlying mechanism of ancestry-assortative mating in human populations.

## Models

### Simulation framework

We performed forward-in-time simulations of admixture with equal contributions from two source populations using SLiM 4.0.1 (Haller et al. 2019; Haller and Messer 2023). We modeled sexually reproducing diploid individuals with 22 independently segregating chromosomes, representing the size distribution of human autosomes (total genome size L=2.88Gb), with a uniform recombination rate $r=1\times 10^{-8}$. We did not model distinct sexes; thus, mating could occur between any two individuals. Unless otherwise noted, there was a single pulse of admixture and no additional contributions from the source populations introduced at later generations. We recorded global and local ancestry for all individuals for each of 50 generations post-admixture, using a constant census population size of *N* = 10,000 individuals per generation. We focus primarily on results from the first 20 generations post-admixture, approximately corresponding to the timing of African-European admixture initiated by the trans-Atlantic slave trade (Zaitlen et al. 2017; Hamid et al. 2021; Korunes et al. 2022; Mas Sandoval et al. 2023; Mooney et al. 2023), with a subset of results over longer time periods in the Supplement.

To investigate the impacts of different mate-choice models, we extended SLiM’s built-in Wright-Fisher framework to include non-random mating using the mateChoice() callback. Specifically, we considered two broad classes of mate-choice models: (1) **ancestry-similarity**, wherein the probability of an individual of ancestry $x_{1}$ mating with an individual of ancestry $x_{i}$ is defined in terms of $|x_{1}-x_{i}|$; and (2) **social group**, wherein the probability of an individual belonging to social group $s_{1}$ mating with an individual belonging to social group $s_{i}$ is defined by whether or not $s_{1}=s_{i}$.

**Figure 1.**
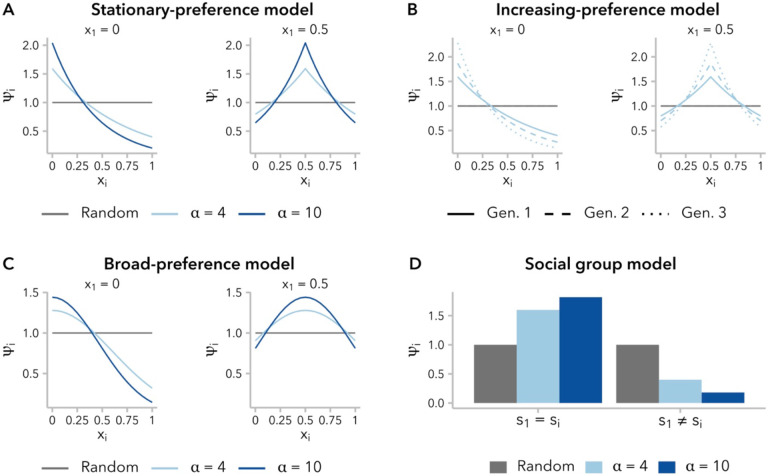
Four functions for defining the sampling weight, $\psi_{i}$, assigned to individual i as a potential mate for a focal individual, based on global ancestry proportion $x_{i}$ (**A-C**) or social group $s_{i}$ (**D**). For each mating event, mate 1 was uniformly sampled from the population, and mate 2 was chosen by weighted sampling. **(A)** The stationary-preference variant of the ancestry-similarity model defines $\psi_{i}$ as a function of the difference in ancestry proportion between individuals, $|x_{1}-x_{i}|$. **(B)** Under the increasing-preference variant of the mate-preference model, the value of $\psi_{i}$ is scaled to the variance in ancestry proportion across all potential mates, resulting in increasing choosiness over time. $\psi_{i}$ values are shown for the first three generations post-admixture for α=4. **(C)** Under the broad-preference variant of the ancestry-similarity model, $\psi_{i}$ follows the probability density function of a normal distribution with mean $x_{1}$. **(D)** Under the social group model, $\psi_{i}$ can take one of two discrete values, determined by whether a potential mate belongs to the same social group as the focal individual ($s_{1}=s_{i}$) or to the other social group ($s_{1}\neq s_{i}$).

Under each model, we constructed mating pairs. First, mate 1 was uniformly sampled with replacement from the population. Second, each individual in the population was assigned a mating weight $\psi_{i}$, calculated by a mate-choice function (defined below) evaluated at $f(x_{1},x_{i})$ or $f(s_{1},s_{i})$, for the ancestry-similarity and social group models, respectively (Figure 1). Third, mate 2 was sampled with probability proportional to $\psi_{i}$, with incidental selfing explicitly prohibited. Each mating event generated a single individual in the subsequent generation.

### Ancestry-similarity models

Individual-based mate preference models originate in the sexual selection literature, where they were developed for studying the co-evolutionary dynamics of male secondary sex characteristics and female mate choice (Lande 1981; Kirkpatrick 1982; Seger 1985). Generally, under these models, one locus controls male phenotype while a second locus controls female preference for that phenotype. In the speciation literature, an alternative family of models are concerned with assortative mating as a mechanism of sympatric speciation and model mate preference based on phenotypic similarity for some ecologically relevant trait controlled by an arbitrary number of loci (Dieckmann and Doebeli 1999; Burger and Schneider 2006; Pennings et al. 2008; Rettelbach et al. 2013). These “matching rule” models (Kopp et al. 2018) are the basis for our ancestry-similarity model class, wherein individuals preferentially choose mates to maximize similarity in phenotype (*i.e.*, ancestry). Importantly for the models we consider here, these are fixed relative-preference models: a focal individual assigns each potential mate a preference value $\psi_{i}$ that depends only on the potential mate’s phenotype and is density independent (Seger 1985).

Ancestry-similarity models define a mate-choice function $\psi_{i}=f(x_{1},x_{i})$ for calculating the sampling weight assigned to individual i with ancestry proportion $x_{i}$as a potential mate for a focal individual with ancestry proportion $x_{1}$. In the present study, we considered three variants of the ancestry-similarity model identified in a literature search, which differ in the precise definition of the mate-choice function f.

Models of ancestry-based assortative mating typically model $f(x_{1},x_{i})$ as an exponential function, such that $\psi_{i}$ decays exponentially at rate c as $|x_{1}-x_{i}|$ increases. We define,

$$\psi_{i}=e^{-c|x_{1}-x_{i}|}.$$

We refer to this as the **stationary-preference model** (“like-with-like” model in (Muralidhar et al. 2022)): for a given difference in ancestry, the associated value of $\psi_{i}$ does not change over time. It is important to note, however, that the range of $\psi_{i}$ is constrained by $x_{1}$: a focal individual with ancestry $x_{1}=0.5$ is more similar to all potential mates than a focal individual with ancestry $x_{1}=0$, and is therefore less selective in choosing a mate (Figure 1A). Furthermore, without additional migration from the source populations, variance in ancestry within the population decreases over time (Figure S2), meaning that the pool of potential mates is becoming increasingly homogenous and the ratio of $\max(\psi_{i})∕\text{min}\;(\psi_{i})$ approaches 1 for all values of $x_{1}$ (Figure S1A). In other words, all individuals become less selective in choosing mates over successive generations.

One approach to account for the decreased variance in ancestry proportion over time, and thus preserve mate-selectiveness over time, is to scale the exponential decay rate c by the generation-specific population variance in ancestry, $\sigma_{g}^{x}$:

$$\psi_{i}=e^{\frac{-c}{\sigma_{g}^{x}}\;|x_{1}-x_{i}|}.$$

We refer to this as the **increasing-preference model** (equation A2 in (Kim et al. 2021)). Assuming an infinite-sites model, $\sigma_{g}^{x}=2^{-g∕2}$, where g is the number of generations post-admixture (Verdu and Rosenberg 2011), although it has been demonstrated that this variance is greater than $2^{-g∕2}$ when accounting for recombination on finite-length chromosomes, as we are here (Liang and Nielsen 2014). Under this model, as under the stationary-preference model, a focal individual with ancestry $x_{1}=0.5$ is less selective than a focal individual with ancestry $x_{1}=0$ (Figure 1B). However, because $\sigma_{g}^{x}$ decreases over time, there is an increase in mate-selectiveness over successive generations under this model for a given $x_{1}$ (Figure S1B; Figure S3).

We also considered a third ancestry-similarity model, employing a Gaussian – rather than exponential – mate-choice function. This approach is commonly used in models of assortative mating based on a quantitative trait (*e.g.*, (Dieckmann and Doebeli 1999; Pennings et al. 2008; Funk et al. 2021)), although it has not to our knowledge been used to model assortative mating based on ancestry proportion. Under this model, $\psi_{i}$ decreases following a normal distribution as ${\left(x_{1}-x_{i}\right)}^{2}$ increases, with the strength of mate-choice preference set by a fixed parameter, σ:

$$\psi_{i}=\frac{1}{\sigma \sqrt{2\pi }}e^{-\frac{{\left(x_{1}-x_{i}\right)}^{2}}{2\sigma^{2}}}.$$

We refer to this as the **broad-preference model** because $f(x_{1},x_{i})$ decays more gradually with increasing differences in ancestry proportion, relative to the stationary-preference and increasing-preference models (Figure 1C). Thus, a focal individual prefers potential mates with similar ancestry proportion to themself over potential mates with dissimilar ancestry proportion, but smaller differences within the pool of “similar” mates are not weighed strongly. Compared to the other two models, individuals are less selective in choosing mates under the broad-preference model; however, as under the stationary-preference model, they also become less selective over time (Figure S1C; Figure S4).

### Social group model

An alternative class of models considers preferential mating based on a categorical, rather than quantitative, trait. This type of model is often used in the context of interspecific hybrid zones, wherein non-random mating can maintain species boundaries and/or promote hybrid speciation (Melo et al. 2009; Schumer et al. 2017; Powell et al. 2021; Natola et al. 2022; Smadja et al. 2022). Mechanistically, this can be construed as a model of biased mating by species or source population (Goldberg et al. 2020). However, application of this type of model in humans is not straightforward: the source populations in question are not biologically distinct species, but rather labels describing socially defined boundaries. Furthermore, it is unclear how we ought to model the mating behavior of admixed individuals: neither random mating by admixed individuals nor a simple preference of admixed individuals to mate with one another seem likely to produce the observed correlation in global ancestry proportion between mates. Yet, the strongest evidence for ancestry-assortative mating in humans is observed in contexts where both spouses have admixed ancestry (Risch et al. 2009; Zou et al. 2015; Zaitlen et al. 2017; Spear et al. 2020; Avadhanam and Williams 2022; Korunes et al. 2022; Mas Sandoval et al. 2023).

Social identity is an important organizer of pair formation in humans, as evidenced by widespread cultural practices of endogamy based on race, ethnicity, religion, socioeconomic status, and caste. Although distinct from genetic ancestry, social categorizations often interact with ancestry. For instance, skin pigmentation, a prominent contributor to how an individual is racialized, is correlated with West African-related ancestry proportion in multiple populations with recent admixture history (Parra et al. 2003; Shriver et al. 2003; Bonilla et al. 2004; Beleza et al. 2012). Race has also served as the basis for numerous sociolegal barriers to mating across contexts, including anti-miscegenation laws (Browning 1951). This type of interaction opens the possibility that social barriers to mating might be “recorded” in patterns of ancestry-assortative mating detectable by genomic analyses.

We considered a simple **social group model** with two groups, in which a mate-choice function $\psi_{i}=f(s_{1},s_{i})$ determines the sampling weight assigned to individual i belonging to social group $s_{i}$ as a potential mate for a focal individual belonging to social group $s_{1}$ as a function of mating bias parameter α (Figure 1D; Figure S5):

ψi={1−1α+1,s1=si1α+1,s1≠si.}

To generate an association between social group membership and ancestry proportion, we assigned individuals in each source population to the same social group prior to admixture. However, from the onset of population contact, individuals “inherited” their social group membership from the first parent in the mating pair. Thus, individuals whose parents belonged to opposite social groups were equally likely to be assigned to either social group.

Several features distinguish the social group model from the ancestry-similarity models described above, or from one in which individuals use an observable quantitative trait (*e.g.*, skin pigmentation) as a proxy for an unobserved trait (genetic ancestry). First, social group membership is correlated with – but not determined by – genotype. This matters because it has previously been demonstrated that recombination is expected to decouple the relationship between ancestry proportion and a proxy quantitative trait over time, leading to only a transient correlation between the two phenotypes (Kim et al. 2021). Because social group membership is not genetic, it is not impacted by recombination. In addition, individuals are uniformly likely to cross the social group barrier in choosing a mate and do not express relative preferences among potential mates within a social group. Thus, in contrast to an ancestry-similarity model, individuals with intermediate ancestry are not less selective than individuals with more source-like ancestry in choosing a mate.

However, these features of the social group model also mean that the association between ancestry proportion and social group membership is expected to decay over successive generations, and we explored this relationship quantitatively over time.

### Defining a common parameter for model comparison

The mate-choice functions we considered differ in how they parameterize the strength of mating bias; to aid in comparison across models, we define an alternate metric of the strength of mating bias, α, which measures the frequency of mating events between individuals from opposite source populations. Specifically, we define α to be inversely related to the proportion of admixed offspring in the initial generation post-contact, A. We have,

$$\alpha =\frac{1}{A}-1.$$

That is, at time of population contact, individuals are α times more likely choose a mate from within their own source population than from the opposite source population. α must be ≥ 0, with increasing values of α representing an increasing bias toward endogamy. α=0 corresponds to complete disassortative mating (all mating pairs are cross-source population), α∈(0,1) corresponds to negative assortative mating, α=1 corresponds to random mating, and α>1 corresponds to positive assortative mating. Given our focus on mechanisms that might explain empirically observed positive correlations, we focus here on α≥1. While models differ in their dynamics over time, comparing simulations with the same α ensures that the correlation in ancestry between mates at generation 1, $r_{t=1}$, is the same. For instance, for the scenario in which both sources contribute equally to a single-pulse admixture event:

$$r_{t=1}=\frac{0.25-(A∕2)}{0.25}=1-2A=1-2(\frac{1}{\alpha +1}).$$

## Results

### All four models produce a positive correlation in global ancestry between mates, at least transiently

**Figure 2.**
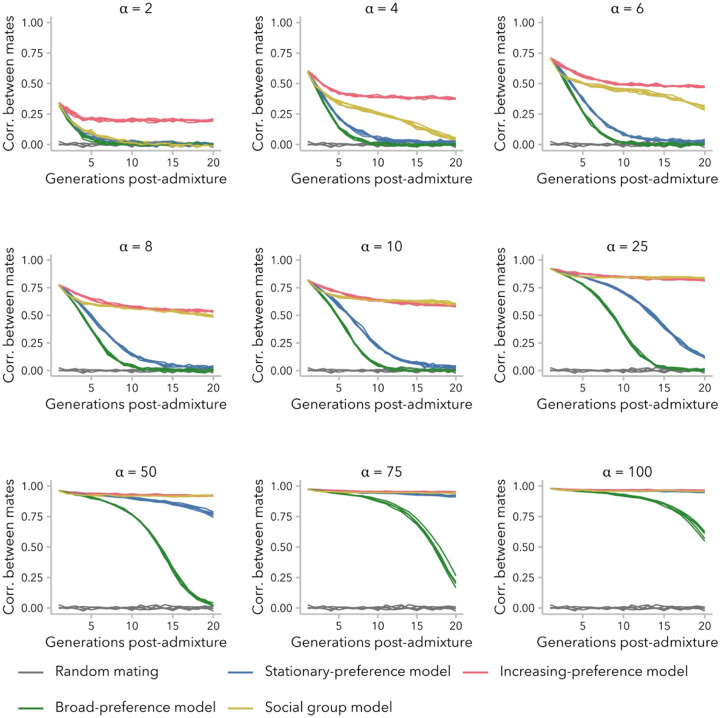
Correlation in ancestry between mates was not constant over time and decayed to near-zero within 20 generations post-admixture in some simulations. Simulations under the stationary-preference (blue) and broad-preference (green) models did not maintain a positive correlation while also allowing for widespread admixture, whereas those under the increasing-preference (pink) and social group (yellow) models did for some α. By definition, simulations performed using the same value of α have the same correlation in generation 1, regardless of model. The same replicate random-mating simulations (α=1; gray) are reproduced in each subplot. See Figure S6.

Ancestry-assortative mating is often inferred from a positive correlation between spouses at single point in time, and statistical methods to account for non-random mating typically generate null models using simulations where potential mate pairs are permuted until the empirically observed correlation is achieved (Zaitlen et al. 2017; Huang et al. 2023; Pfennig and Lachance 2023). This approach assumes that the correlation observed in the present-day samples has remained constant since the start of admixture. Figure 2 plots the Pearson correlation coefficient between mates over time under three variants of the ancestry-similarity model or the social group model, for multiple strengths of assortative mating (values of α) and following a single pulse of admixture. In all scenarios of non-random mating that we considered, we observed positive correlations in ancestry proportion between mates (Figure 2; Figure S6). However, contrary to the assumption that the correlation in ancestry observed in a contemporary sample should be modeled as a fixed parameter, the observed correlation coefficients in our simulations decayed over time. In simulations performed under the increasing-preference model, correlation between mates reached a stable plateau within the first 10 generations post-admixture across values of α (Figure S7A). In contrast, simulations performed under the other two variants of the ancestry-similarity model (stationary-preference or broad-preference) approached correlations of zero within 20 generations post-admixture, unless α was sufficiently large to disrupt the admixture process (Figure S8; Figure S9). Under the social group model, positive correlations between mates could be observed 20 generations post-admixture for α>2, although these correlations decayed to zero on longer time scales (Figure S7B). Thus, not all variants of the ancestry-similarity model generate a correlation in ancestry between mates observable 20 generations post-admixture while also allowing for a large population of individuals within intermediate ancestry. Conversely, results for the social group model demonstrate that even relatively weak social barriers can produce correlations in ancestry between mates on the timescale of tens of generations, even as the association between social group membership and ancestry proportion diminishes over time (Figure S10).

To understand mechanisms that could potentially explain the correlations between spouses observed in empirical data, in the following sections we will focus on the two models that produce measurable positive correlations between mates 20 generations post-admixture: the increasing-preference variant of the ancestry-similarity model, and the social group model. Specifically, we compare pairs of simulations under these models that produced the same correlation coefficient at generation 20.

### Similar correlation coefficients may be produced by different underlying distributions of ancestry proportion

Though commonly used for empirical genetic studies, the correlation between mating pairs is not particularly informative about the underlying distribution of global ancestry proportion, across individuals or mating pairs. We next extended our perspective beyond correlation coefficients to broader patterns of ancestry proportion under the increasing-preference and social group models.

Over time, the distribution of ancestry proportion x shifts from two discrete populations to a continuous distribution of intermediate values. Random mating produces a unimodal distribution of ancestry proportion, here seen from generation 2; under ancestry-biased mating, individuals with more extreme values of x are likely to mate with one another, meaning that we expect the shift from bimodal to unimodal distribution to be delayed. However, the timing of this shift is delayed more under the social group model than under the increasing-preference model for the same α (Figure S11). Furthermore, this difference in the distribution of ancestry proportion under the two models is observed even when comparing pairs of simulations that have similar correlation coefficients.

Specifically, we analyzed the distributions of global ancestry within the admixed population at generation 20, approximating a common time since admixture for multiple human populations with African and European ancestry (Zaitlen et al. 2017; Hamid et al. 2021; Korunes et al. 2022; Mas Sandoval et al. 2023; Mooney et al. 2023). At g=20, we found a unimodal distribution of ancestry proportion for simulations under the increasing-preference model for all values of α, whereas the social group model produced a bimodal distribution for simulations with α≥7. For simulations under the social group model with α<7, ancestry proportion was unimodal but with higher variance than simulations under the increasing-preference model with the same correlation between mates (Figure S12).

**Figure 3.**
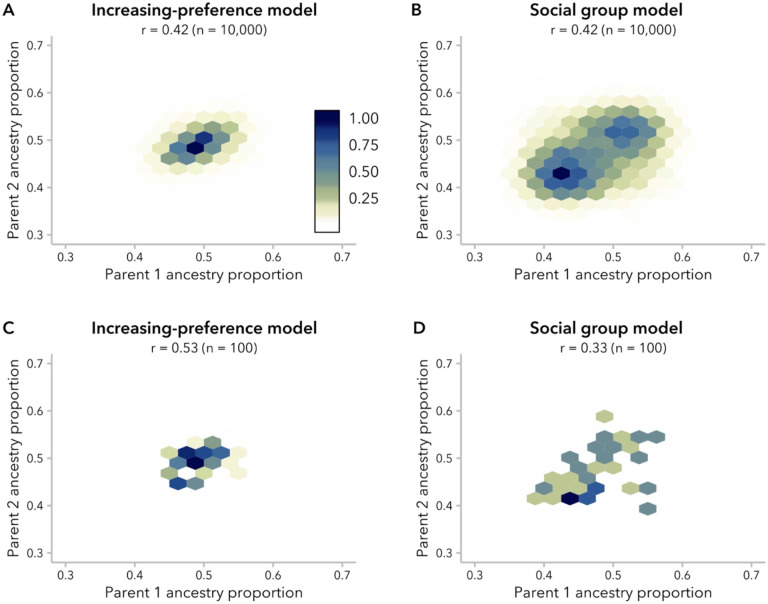
Simulations under the increasing-preference (α=5) and social group (α=7) models produced similar correlation coefficients with different underlying mating structure. “Hexbin” plots represent correlation in ancestry proportion between the two parents of individuals in generation 20, with each hexagon corresponding to a bin of 0.025 ancestry proportion units. The density of mating pairs in each ancestry bin is represented by the color, with darker colors representing higher density. (Density values are scaled to a maximum of 1). **(A, B)** Under the increasing-preference model, mating pairs cluster around a single bin of maximum density, whereas under the social group model, there are two centers of density. All 10,000 individuals are shown. **(C, D)** Density plots are suggestive of differences in mating structure between models, even with a sample size of 100 individuals.

The difference in the distribution of ancestry proportion at generation 20 between the two models could also be observed in plotting the correlation between mating pairs (Figure 3A-B): under the increasing-preference model, there was a single cluster of mating pairs with the highest density around x=0.5, corresponding to the original admixture contributions. In contrast, under the social group model, we were able to distinguish two clusters of mating pairs, symmetric around the population mean. These clusters were apparent when the correlation in ancestry between mates was ≥ 0.4 (Figure S13). Thus, variants of both the ancestry-similarity and the social group model can produce similar correlations, but these models may be distinguishable by examining underlying structure in mating pairs, especially when the correlation in ancestry between mates is sufficiently strong.

Given that evidence for ancestry-assortative mating is often assessed in much smaller empirical datasets, we also examined whether these differences in mating structure could be observed in a subsample of our simulation population (n=100 individuals). Correlation in ancestry between mates is typically visualized as a dot-plot (*e.g.*, (Zou et al. 2015; Korunes et al. 2022)); we found that the two models looked similar when taking this approach (Figure S14). However, even with 100 individuals, density plots were suggestive of discrete clusters of mate pairs in simulations under the social group model with high α, and thus high correlation in ancestry between mates (Figure 3C-D; Figure S15).

### Variance in ancestry proportion determines whether or not correlation in ancestry between mates is maintained over time

Although all of the models we consider define functions for non-random mate choice, we observed in Figure 2 that for the stationary-preference and broad-preference models, the correlation in ancestry proportion between mates either decayed to zero within the first 20 generations post-admixture (α≤10) or permitted very limited admixture (α≥25). To understand why this correlation approaches zero under these models, but not under the increasing-preference and social group models, we compared the population variance in ancestry proportion over time under each model. Intuitively, variance is necessary for observing non-random mating: if all potential mates are identical, mate choice will appear arbitrary. Thus, we would expect our simulations to become indistinguishable from random mating, even though we are modeling biased mating, if variance in ancestry proportion becomes sufficiently small.

**Figure 4.**
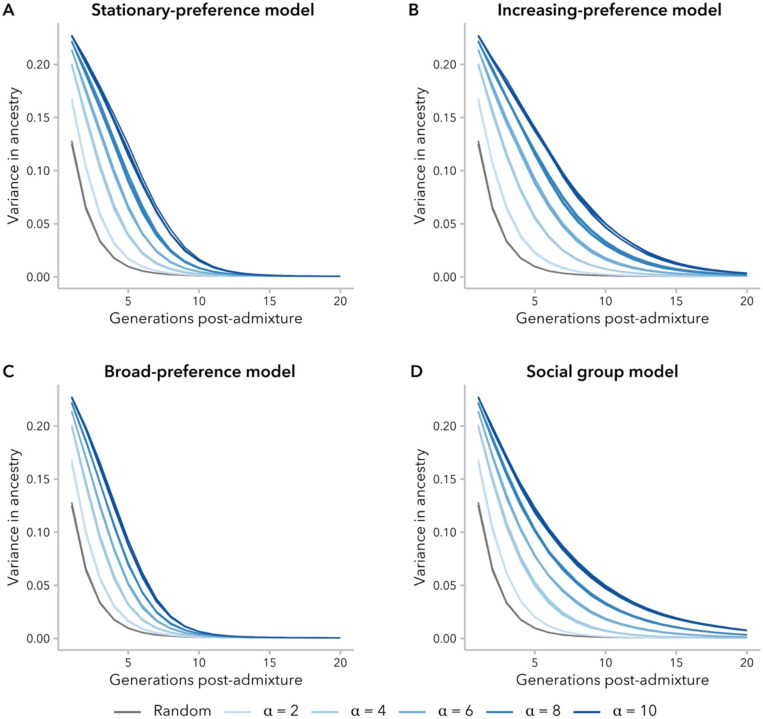
Variance in ancestry proportion decayed to zero over time for a single-pulse admixture scenario, but the decay is slower for larger values of α. At twenty generations post-admixture, only simulations under the increasing-preference and social group functions **(B, D)** had a greater variance than the random-mating control simulations.

Consistent with prior analytic models, non-random mating resulted in higher variance in ancestry proportion relative to random mating at a given generation, regardless of mating model (Wright 1921; Verdu and Rosenberg 2011; Liang and Nielsen 2014; Zaitlen et al. 2017; Goldberg et al. 2020), and variance decayed more slowly when α was larger (Figure 4). However, variance in ancestry proportion for simulations under the stationary-preference and broad-preference models approached to zero within ~15 generations post-admixture under our set of parameters. This timing corresponds to the decay to zero in correlation between mates observed in Figure 2 under these two models (Figure S16). In contrast, for the increasing-preference and social group models, which sustained a correlation between mates over 20 generations, variance in ancestry proportion was greater than for random mating at each time point (all α for increasing preference; α>2 for social group).

To quantitatively assess whether the decay in correlation between mates meant that mating was becoming effectively random over time, we performed permutation tests comparing the correlation coefficient for the observed mating pairs to randomized mating pairs (1,000 permutations). We found that this was not necessarily the case: for α=10, correlation between mates under the stationary-preference model was significantly different from random 20 generations post-admixture even in a simulation for which r<0.05. However, under the broad-preference model, the correlation between mates for α=10 was indistinguishable from random by around 10 generations post-admixture (Figure S17).

Given the limited information from a single summary statistic, the correlation coefficient of global ancestry for mating pairs, we next explored an alternative metric of biased mating: the absolute difference in ancestry proportion between mate pairs, $\Delta_{x}=|x_{1}-x_{i}|$. A small mean value of $\Delta_{x}$ indicates a tendency for individuals to have similar ancestry proportion to their mates. Importantly, however, the value of $\Delta_{x}$ cannot be interpreted outside the context of the population-specific variance in ancestry; ${\bar{\Delta }}_{x}$ is smaller under random mating than for a simulation with α=10 because the mean difference between any pair of individuals is smaller (Figure S18). Permutation tests for $\Delta_{x}$ were consistent with those for the correlation coefficient: the stationary-preference model was significantly different from random mating at generation 20 for α>5 (*p* = 0.001), whereas the broad-preference model became “random-like” within the first 5-10 generations post-admixture (Figure S19).

To compare between models, we devised a statistic that we named expressed mating bias, B, to quantify how much smaller ${\bar{\Delta }}_{x}$ is than expected given the simulation- and generation-specific variance of the population from mate pairs are drawn. Specifically, we define B as 1 less the ratio of ${\bar{\Delta }}_{observed}$ to the mean ${\bar{\Delta }}_{permuted}$ across permutations:

B=1−∕Δ¯¯permutedΔ¯observed.

We define B in this manner so that B=0 when mating is random (${\bar{\Delta }}_{observed}={\bar{\bar{\Delta }}}_{permuted}$) and B=1 when mating is so biased that no admixture is permitted (${\bar{\Delta }}_{observed}=0$). Within this range, increasing B corresponds to more biased mating.

**Figure 5.**
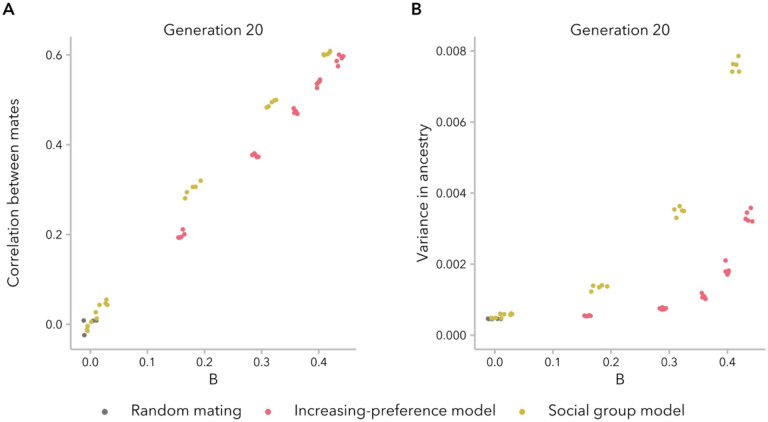
Variance in ancestry proportion within the population constrains the possibility of observing a correlation in ancestry between mates. **(A)** Correlation coefficients are correlated with an alternative metric of assortative mating, expressed mating bias B, which accounts for variance. Each dot represents one simulation. **(B)** Variance in ancestry increases with increasing B, but not uniformly across models. A given value of B corresponds to lower variance in ancestry under the increasing-preference model, emphasizing that biased mating is maintained in these simulations in the face of low variance.

We observed a similar relationship between the observed correlation between mates and B across models and time points, confirming that correlation coefficient largely reflects the degree of similarity between mates relative to the possible alternative pairs (Figure 5A; Figure S20). We observe a different trend when considering ${\bar{\Delta }}_{observed}$: individuals are less similar to their mates (${\bar{\Delta }}_{observed}$ is greater) for the same correlation under the social group model compared to the increasing-preference model (Figure S21). This underscores the importance of accounting for bias in mate choice *relative to variance*.

As expected, higher variance in ancestry proportion corresponded to higher B across models. However, for the same value of B, simulations under the increasing-preference model had lower variance than those under the social group model (Figure 5B). This emphasizes a key distinction between the two models: the social group model sustains high correlation in ancestry between mates over tens of generations by maintaining higher variance in the population, whereas the increasing-preference model instead compensates for very low variance by explicitly scaling the mate-choice parameter c by the variance, increasing the strength of preference.

### Ancestry-assortative mating produced in an excess of long local-ancestry tracts relative to the exponential distribution under both model classes

Thus far, we have focused on the relationship between mating model and the distribution of global ancestry proportion. We next considered the impact of model choice on local-ancestry tract length. Local-ancestry tracts are used in population-genetic methods to infer demographic parameters, including the time since the onset of admixture (Moorjani et al. 2011; Gravel 2012; Loh et al. 2013; Hellenthal et al. 2014), and prior work has shown that non-random mating results in longer local-ancestry tracts, leading to underestimation of this parameter (Zaitlen et al. 2017; Korunes et al. 2022). Thus, some methods to correct for the effects of assortative mating attempt to recapitulate local-ancestry tract dynamics by matching the empirical correlation between spouses (Zaitlen et al. 2017).

**Figure 6.**
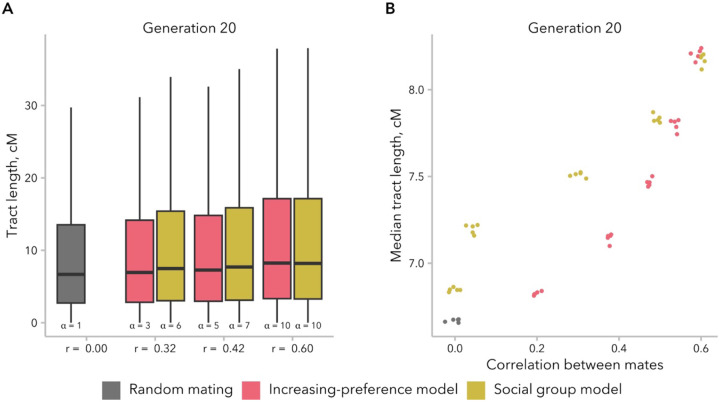
Local-ancestry tract length distributions were similar for simulations under the increasing-preference and social group models, when comparing simulations with similar correlation in ancestry between mates. **(A)** The distribution of local-ancestry tract lengths at generation 20 is shown for three representative pairs of simulations, compared to a random mating control. The y-axis is truncated at the top whisker (75^th^ percentile plus 1.5 times the inter-quartile range). **(B)** Median local-ancestry tract length was similar across models and values of α. However, the median tract length tended to be longer for simulations under the social group model relative to those under the increasing-preference model when comparing simulations with the same correlation in ancestry between mates. Each dot represents one simulation (5 replicates per α).

We first compared the median local-ancestry tract length 20 generations post-admixture in simulations under the increasing-preference and social group models, matched for correlation between mates. Differences in median tract length were modest when comparing across α values within a single model, between these two mate-choice models, and between both models and random mating (Figure 6A). However, we did observe across all four mate-choice models that larger values of α were associated with longer median tract lengths at generation 20 (Figure S22). Furthermore, under the increasing-preference and social group models — the two models under which there was still a positive correlation between mates at generation 20 — greater correlations between mates were also associated with longer median tract lengths. Additionally, for a given correlation between mates, median local tract length tended to be longest under the social group model (Figure 6B).

**Figure 7.**
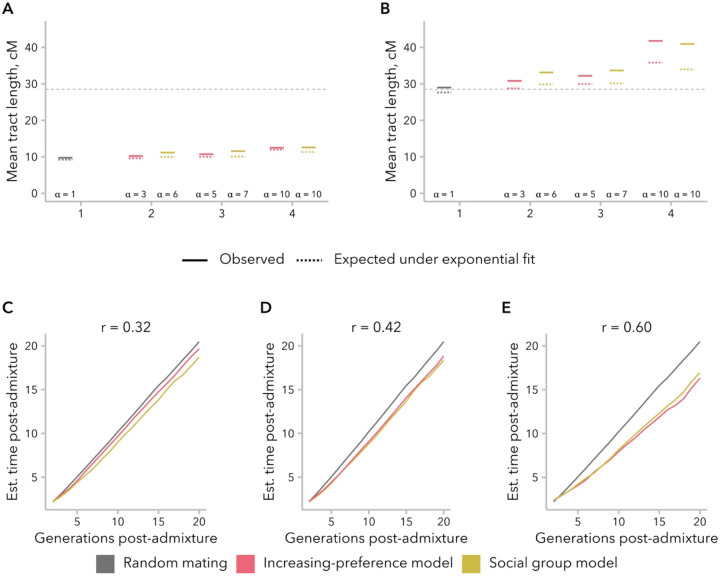
Non-random mating led to underestimation of the time since admixture. For each simulation, an exponential model was fit to the local-ancestry tract length distribution, with decay rate λ=g×m(g: generations since admixture; m: admixture contribution). **(A)** The observed local-ancestry tract length at generation 20 was similar to the mean tract length expected under the exponential distribution $\left(\frac{1}{\lambda }\right)$, indicating a good fit to the data. The dashed line indicates the expected mean tract length for the true values of g and m. **(B)** The observed 95^th^ percentile of the local-ancestry tract length distribution was longer than expected under the exponential fit: $Q_{95}=\frac{-\text{ln}(1-0.95)}{\lambda }$, particularly when the correlation between mates is high. The dashed line indicates the expected 95^th^ percentile tract length for the true values of g and m. **(C)** Estimates for the time since admixture based on the exponential fit to the local-ancestry tract distribution were similar between the increasing-preference and social group models. As expected, time since admixture was underestimated when mating was biased, and the discrepancy was greater when the correlation in ancestry between mates was greater.

To directly test the effects of the increasing-preference and social group models on downstream inference of admixture timing, we fit an exponential model to the local-ancestry tract length distribution to infer the time since a single admixture event (Equation 1 from (Gravel 2012)). We observed that the empirical distribution had a heavier right tail than the exponential fit, particularly as the correlation between mates increased (Figure 7A, B). Although slight, this mismatch in distribution shape may prove useful as an indication in empirical data that the effects of non-random mating should be taken into consideration, although it may also be confused for evidence of continuous migration.

As expected, we underestimated the time since admixture when using local-ancestry tract length distributions, and the discrepancy between the truth and our estimate increased as the correlation in ancestry between mates increased (Figure 7C). Intriguingly, we found that this discrepancy was established in early generations and grew relatively slowly over time, suggesting that there might be a plateauing of the effect on longer timescales (Figure S23). For instance, time since admixture was underestimated by an average of 2.25 generations for 20 generations, and 4.01 generations for 50 generations. As a result, we observe parameter-inference bias of a similar magnitude under models for which a correlation in ancestry is no longer observed (Figure S24).

### The effects of continuous migration on the correlation in ancestry between mates differed between models and values of α

Realistic models of human admixture likely include more complex dynamics than a single pulse of admixture followed by complete isolation from the source populations, as we have modeled here. To begin to explore the behavior of the increasing-preference and social group models under more complex demographic scenarios, we examined the trajectory of the correlation in ancestry between mates over time in a scenario with continuous migration, wherein 1% of the population in each generation was replaced with migrants from the source populations. Relative to the single-pulse admixture scenario, we might expect that the correlation between mates would be decreased (for the same generation and α) because new migrants have fewer potential mates with similar ancestry proportion to choose from compared to individuals born in the admixed population. Furthermore, in each successive generation, variance in ancestry proportion decreases for the admixed population (Figure 4), meaning that new migrants have increasingly dissimilar ancestry proportion to the average potential mate. On the other hand, mating events between two migrants from the same source population will increase the correlation coefficient.

**Figure 8.**
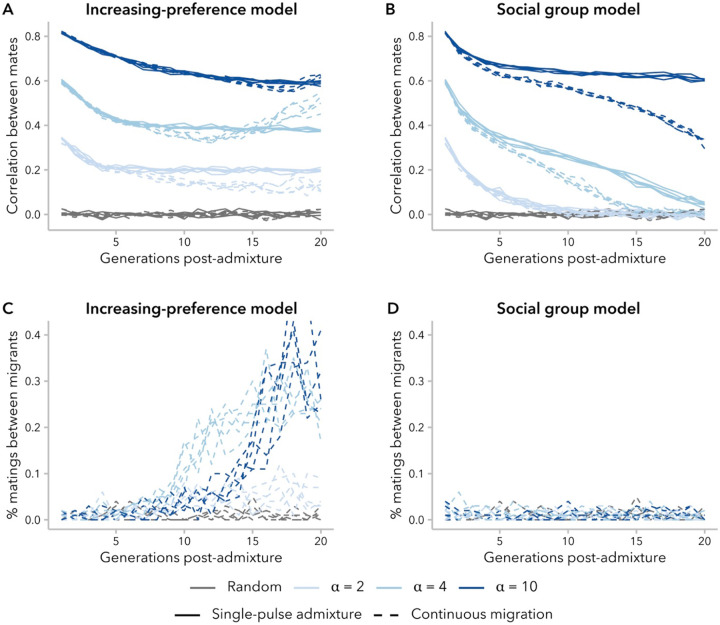
The effects of continuous migration differed between mate-choice models, driven by the percent of mating events between new migrants. **(A, B)** Correlation in ancestry between mates increased over time in simulations under the increasing-preference model with continuous migration, for some α. This behavior was not observed in any simulations without migration (see Figure 2) or under the social group model with continuous migration. **(C)** Under the increasing-preference model with continuous migration, mating between migrants was increasingly prevalent over time, and far more frequent than expected under random mating. **(D)** Mating between migrants under the social group model with continuous migration did not occur more often than expected by chance.

We found that the balance between these countervailing effects on the correlation between mates differed between models. Under the increasing-preference model, the correlation coefficient initially decreased over time, similar to the scenario with single-pulse admixture. However, for simulations with α≥4, there was a subsequent increase in correlation over time, resulting in a greater correlation coefficient at generation 20 in the scenario with migration than the one without migration (Figure 8A). As noted above, the increasing-preference model compensates for decreasing variance by increasing mate selectiveness over successive generations (Figure 5B). Consistent with this explanation for the difference between the single-pulse admixture and continuous migration simulations, we observed that the proportion of mating events between migrants increased over time in these simulations (Figure 8C). Additionally, under the other two ancestry-similarity models, which do not compensate for the decreased variance over time, we observed that the prevalence of mating events between migrants was constant and that the correlation between mates was similar with or without migration for a given generation and α (Figure S25; Figure S26; Figure S27).

In contrast, the social group model neither takes into consideration similarity in ancestry proportion, nor increases the strength of mating bias over time. Thus, for simulations under this model, new migrants are equally likely to mate with anyone within their social group regardless of ancestry proportion; as expected, we observed a constant prevalence in mating events between migrants (Figure 8D). Additionally, because variance in ancestry decreases over time, migrants are more and more dissimilar from potential mates. As a result, continuous migration always decreased the correlation in ancestry between mates under this model relative to the single-pulse admixture scenario, controlling for generation and α (Figure 8C).

## Discussion

While it is appreciated that humans, like individuals in many other natural populations, do not choose mates at random, population-genetic theory and methods to account for assortative mating in empirical data remain largely underdeveloped. In the context of ancestry-assortative mating, extension of existing theory on how assortative mating shapes the expected distribution of phenotypes in a population (Wright 1921; Norris et al. 2019; Kim et al. 2021; Border et al. 2022; Muralidhar et al. 2022; Horwitz et al. 2023) is made difficult by ambiguity about what the relevant phenotype is. Furthermore, global ancestry proportion is an unusual quantitative phenotype, in that its trait value is determined by every locus in the genome, meaning that the entire null distribution of any parameter of interest is likely to be affected. Simulations of ancestry-assortative mating that accurately recapitulate key summaries of empirical data are crucial to unraveling these impacts. Here, we considered two related prerequisite questions: first, are there multiple mechanisms of mate choice compatible with the observed correlation in global ancestry proportion in human populations? Second, does the choice of a particular mathematical function for defining biased mating meaningfully impact the conclusions drawn from the resulting simulations? We compared four models, including one that considers social groups rather than quantitative similarity in ancestry as the mechanism of mate choice, to better understand how we ought to think about modeling and correcting for ancestry-assortative mating.

We turn first to assumptions commonly made by statistical genetics methods (Zaitlen et al. 2017; Huang et al. 2023; Pfennig and Lachance 2023), which focus on the observed empirical correlation in ancestry between mates: namely, that this correlation is constant over time and that parameters of interest are comparable between empirical data and simulated data with the same correlation coefficient. In our simulations, we find that correlation coefficients are not stable over time and decay toward zero under most models (Figure 2). Thus, assuming that bias in mating is constant over time, the correlation between spouses observed in a contemporary sample is likely smaller than it was in previous generations. In addition, we find that multiple models can produce the same correlation between mates while differing in the underlying mating structure (Figure 3), average difference in ancestry between mates (Figure 5), and the right tail of the local-ancestry tract length distribution (Figure 6). However, we find that the effects of these differences between models on estimated time since admixture are likely to be small, likely because the effects on mean tract length are similar (Figure 7).

Prior studies that have modeled biased-mate choice to develop theory about assortative mating (Kim et al. 2021; Muralidhar et al. 2022) have, sometimes implicitly, assumed that these models are sufficient to generate a correlation in ancestry. We find that this assumption is not true for all models, bias parameters, and generations. Specifically, variance in ancestry proportion plays an essential role in determining whether a correlation between mates can be observed and, furthermore, whether mating is effectively random (Figure 4; Figure 5). This approach has also prioritized the role of individual mate-choice preference for similarity in ancestry ($|x_{1}-x_{i}|$). In humans particularly, there is a wide array of social science research to support the role of sociological factors in mating outcomes. To wit, we found that a simplistic model imposing a barrier to mating between two social groups was sufficient to generate signatures of ancestry-assortative mating that could be observed for 20-50 generations post-admixture. To our knowledge, this type of model has not been used before to model ancestry-assortative mating in admixed populations but may be representative of how social categories like race, ethnicity, and socioeconomic status influence spouse choice.

The four models that we consider in the present study are not the only possible models, but rather represent two classes of model worthy of further exploration: ancestry-similarity and social group models. For ancestry-similarity models, we have demonstrated that not all variants of this model type can sustain sufficiently high levels of variance to continue to observe positive correlations in ancestry (at least, without additional in-migration events). The increasing-preference model compensates for this decay in variance by increasing the choosiness of individuals. While this is effective in maintaining the correlation in ancestry, the current approach is likely making individuals unrealistically attuned to small differences between mates (Figure S2B). This overcompensation is heightened in the continuous-migration scenario, leading to unexpected (and undesirable) behavior (Figure 8). Future development of ancestry-similarity models should consider alternative approaches to tune the selectiveness of individuals over time. For instance, we might want to model a constant degree of bias (*i.e.*, $\max(\psi_{i})∕\text{min}\;(\psi_{i})$) over time.

In including the social group model in this study, we aimed only to demonstrate that social barriers can mediate ancestry-assortative mating, even when individuals do not directly use ancestry information to make mating decisions. To that end, we designed a proof-of-concept version of this model class, which is likely overly simplistic for drawing conclusions about real-world human populations. Future development of this class could include greater social complexity; for instance, more than two social groups, alternative rules for how individuals “inherit” their social group membership, and asymmetric barriers between groups. Each of these added layers could be implemented in different ways. For example, in a simulation with three groups, barriers might be more permeable between some pairs of groups than others.

A growing body of research suggests that patterns of assortative mating is not stable over time (e.g., (Mare 1991; Sunde et al. 2024)). Under an ancestry-similarity model, this could reflect changes in preference over time, potentially to account for decreasing differences between potential mates (as discussed above). Under a social group model, this could reflect changes in social mobility or acceptance of inter-group mating. Thus, modeling this type of change over time would be an interesting future direction for both classes of model, although it remains outside the scope of the present work.

Our results lead us to make several recommendations for future empirical studies of ancestry-assortative mating in humans. First, a correlation in ancestry between spouses can reflect multiple mechanisms of mate choice and should not be interpreted as unequivocal evidence of a preference for mates with similar ancestry proportion. Second, there are many different population structures that can give rise to the same Pearson correlation coefficient, and visualization of these structures may help to disambiguate these possibilities. In particular, rather than the more commonly used dot-plot, we suggest that density plots be used as an additional, often more informative, visualization tool. Third, difference in ancestry between mates (${\bar{\Delta }}_{obs}$) was informative about differences between simulations with similar correlation coefficients. Importantly, this metric should only be interpreted relative to permutations of the data because its value is constrained by the variance in ancestry present in the dataset.

As with any simulation study, our results are limited by what we have elected to model or not model. Of particular note, we have focused on a single pulse of admixture, turning to a continuous-migration scenario only to highlight how one of our models behaves unexpectedly. More realistic models of human populations almost certainly involve much more complex migration dynamics, including multiple pulses of migration, differing contributions from source populations, changes in population size, asymmetries in mate preferences and in the ability of individuals to enact their mate choices, and multi-way admixture. Inclusion of at least some of these additional factors into future models is likely to impact the results; however, each additional factor drastically expands the range of possible implementations and parameter values. As such, these more complex models are likely to be more useful when targeted to matching the parameters of a particular population of interest whose history is well understood and less well suited to a general exploration of parameter space.

## Supplementary Material

Supplement 1

## Data Availability

All scripts used for simulations, analyses, and figures is available on GitHub at: https://github.com/agoldberglab/ancestry-assortative-mating-simulation.
