# Supplementary material for: Differentiating mechanism from outcome for ancestry-assortative mating in admixed human populations
Source: bioRxiv. 2024 Jun 6:2024.06.06.597727. Preprint. [Version 1] doi: 10.1101/2024.06.06.597727 (PMC11185628; doi:10.1101/2024.06.06.597727)
Supplement: Supplementary file 1 [file media-1.pdf]

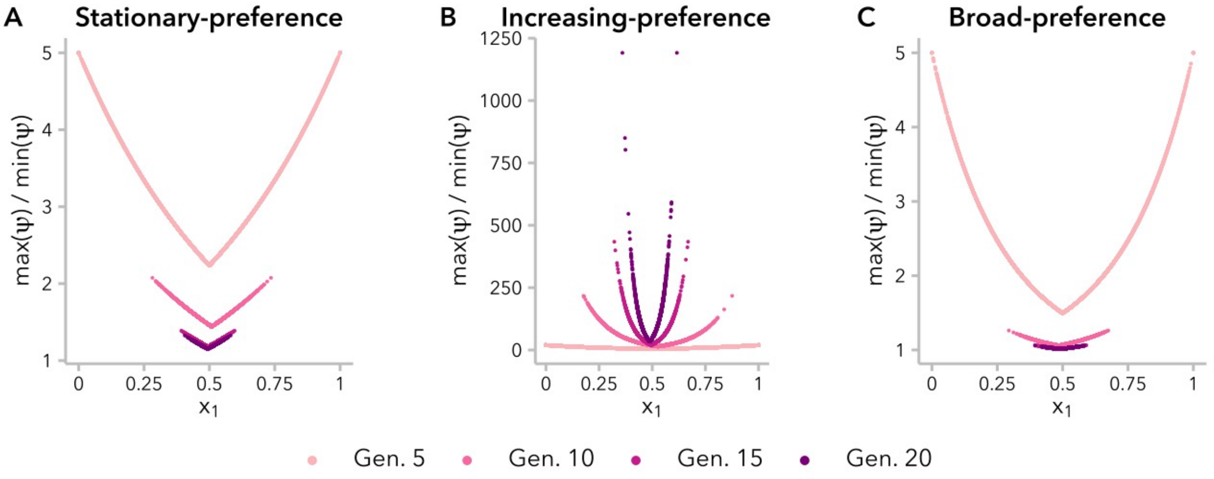

Figure S1. Individuals differ in "choosiness" – quantified as the ratio of $max(\psi)\ /min\ (\psi)$ – based on their own ancestry proportion, x_1, and on the relative homogeneity of potential mates. $\alpha = 5$ is shown for all three models. **(A)** Under the stationary-preference model, $max(\psi)\ /min\ (\psi)$ approaches 1 for all values of x_1 over time. **(B)** Under the increasing-preference model, $max(\psi)\ /min\ (\psi)$ increases exponentially over each successive generation, resulting in increased choosiness over time. **(C)** Under the broad-preference model, $max(\psi)\ /min\ (\psi)$ approaches 1 for all values of x_1 more quickly than under the stationary-preference model.

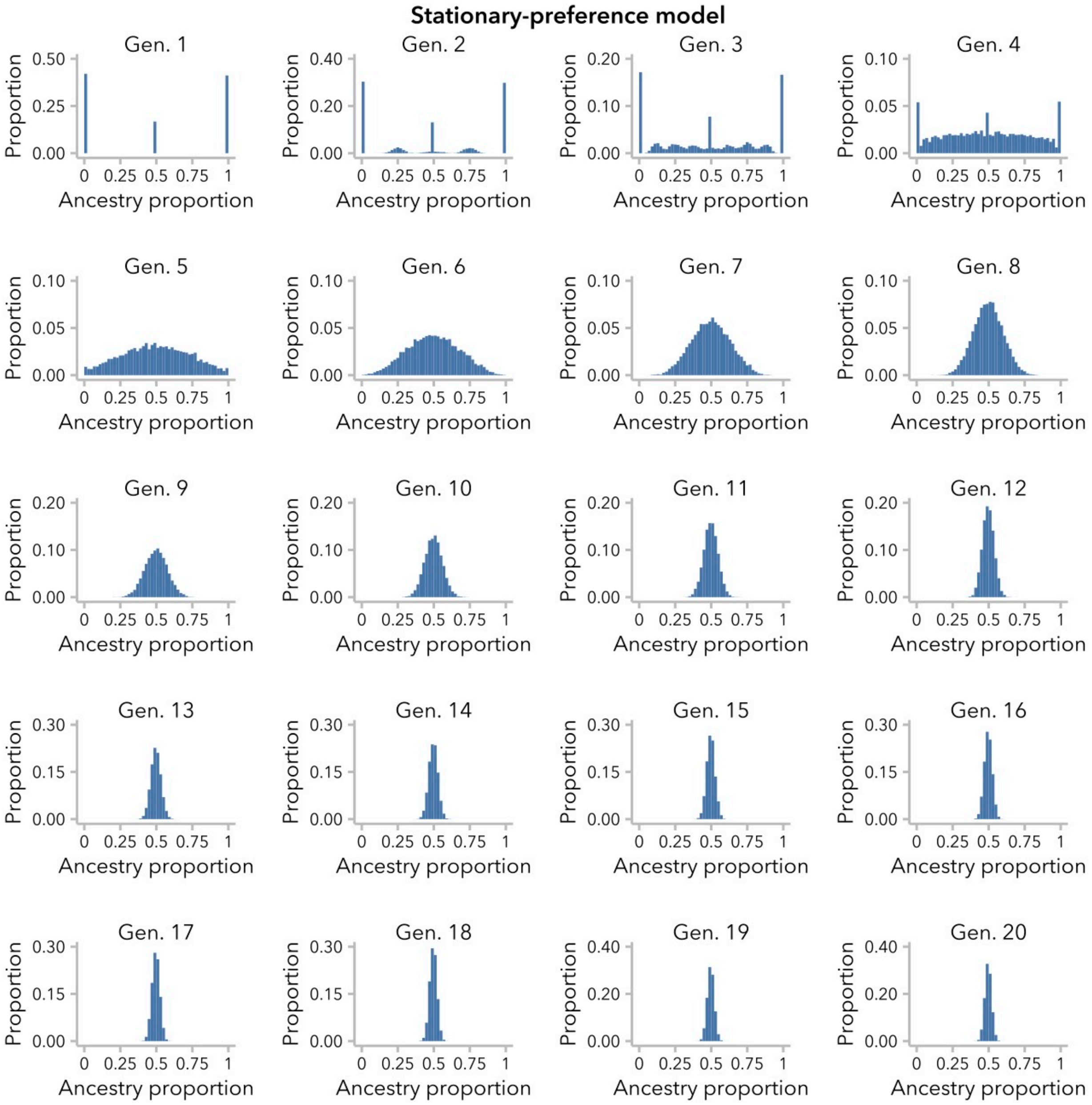

Figure S2. Under the stationary-preference model, variance in ancestry within the population decreases as admixture proceeds. The distribution of ancestry proportion is plotted for the first 20 generations post-admixture for $\alpha = 5$.

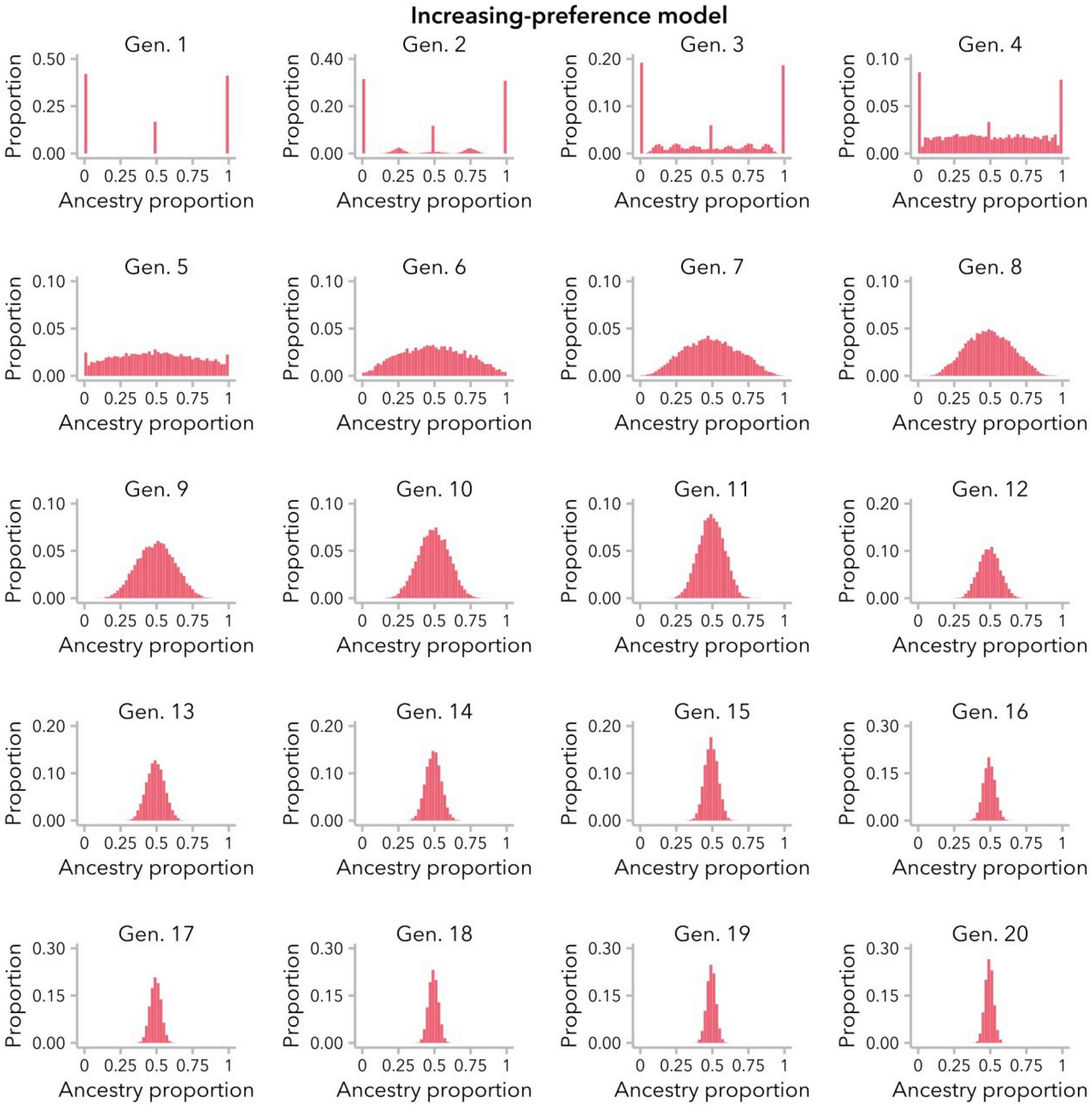

Figure S3. Under the increasing-preference model, variance in ancestry within the population decreases as admixture proceeds. The distribution of ancestry proportion is plotted for the first 20 generations post-admixture for $\alpha = 5$. To compensate for the decreased variance over time, the mate-choice parameter is scaled by the generation-specific variance σ_g^x, meaning that the ratio of $max(\psi)\ /min\ (\psi)$ increases over time (see **Figure S1B**).

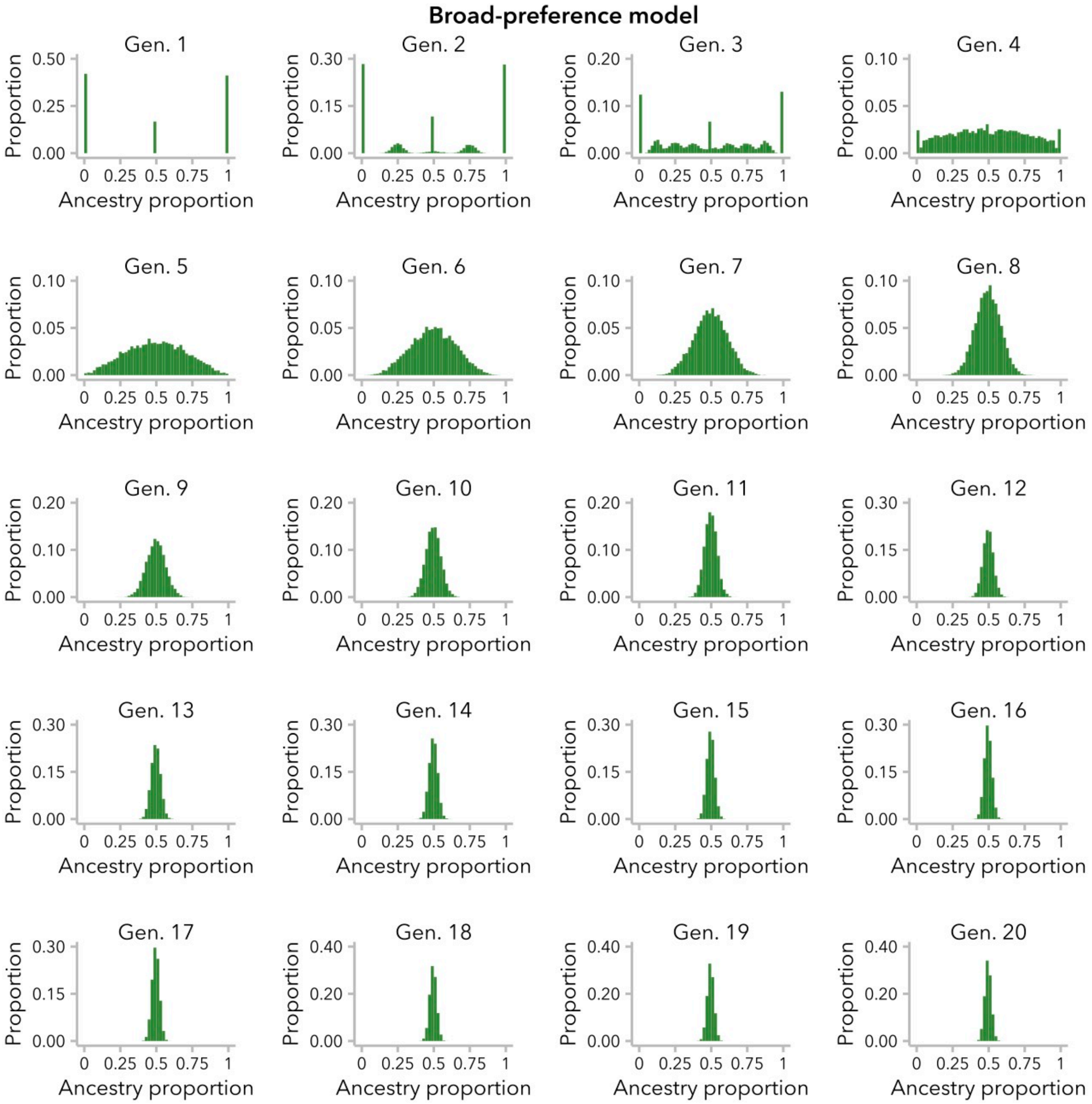

Figure S4. Under the broad-preference model, variance in ancestry within the population decreases as admixture proceeds. The distribution of ancestry proportion is plotted for the first 20 generations post-admixture for $\alpha = 5$.

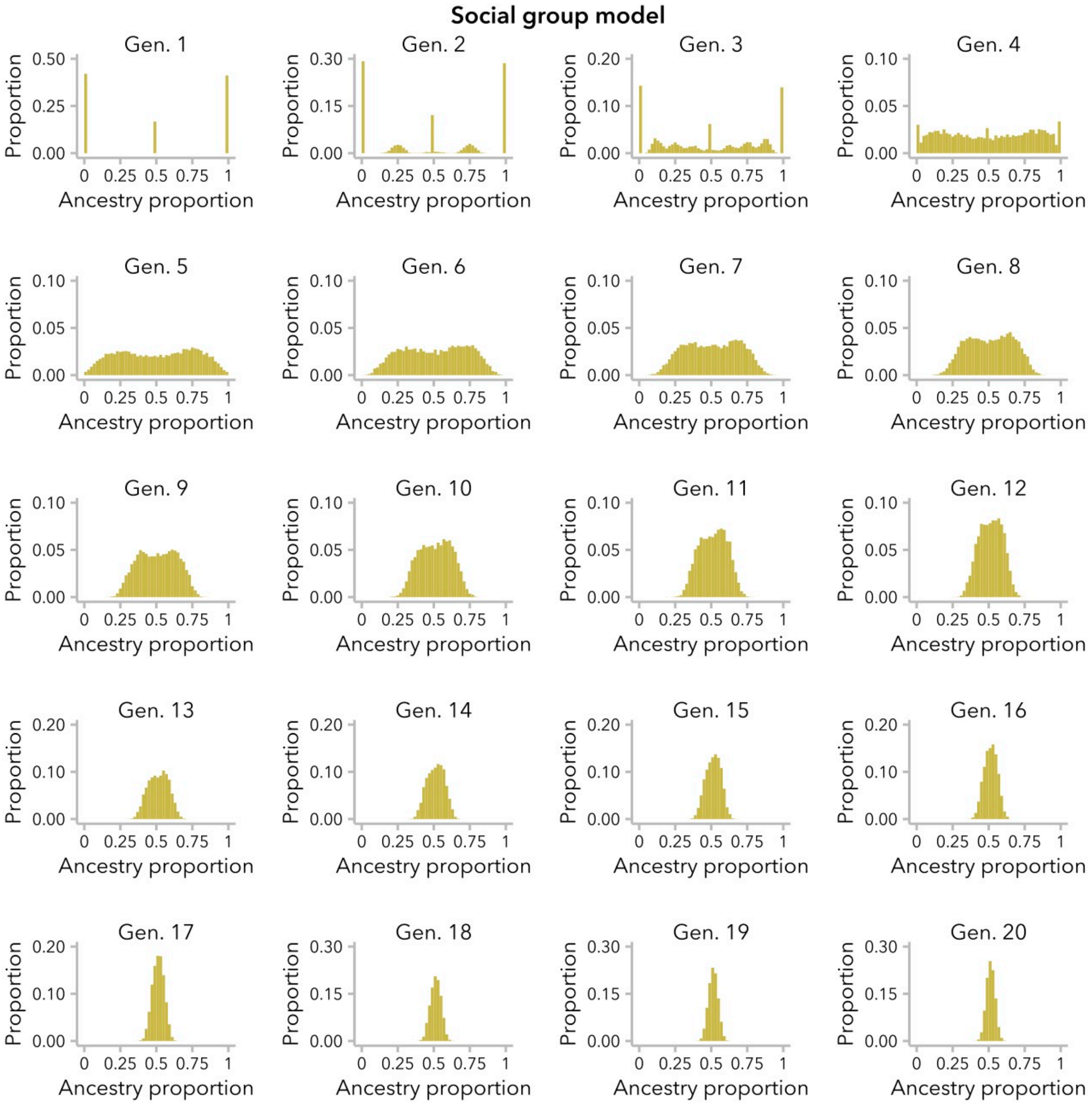

Figure S5. Under the social group model, variance in ancestry within the population decreases as admixture proceeds. The distribution of ancestry proportion is plotted for the first 20 generations post-admixture for $\alpha = 5$.

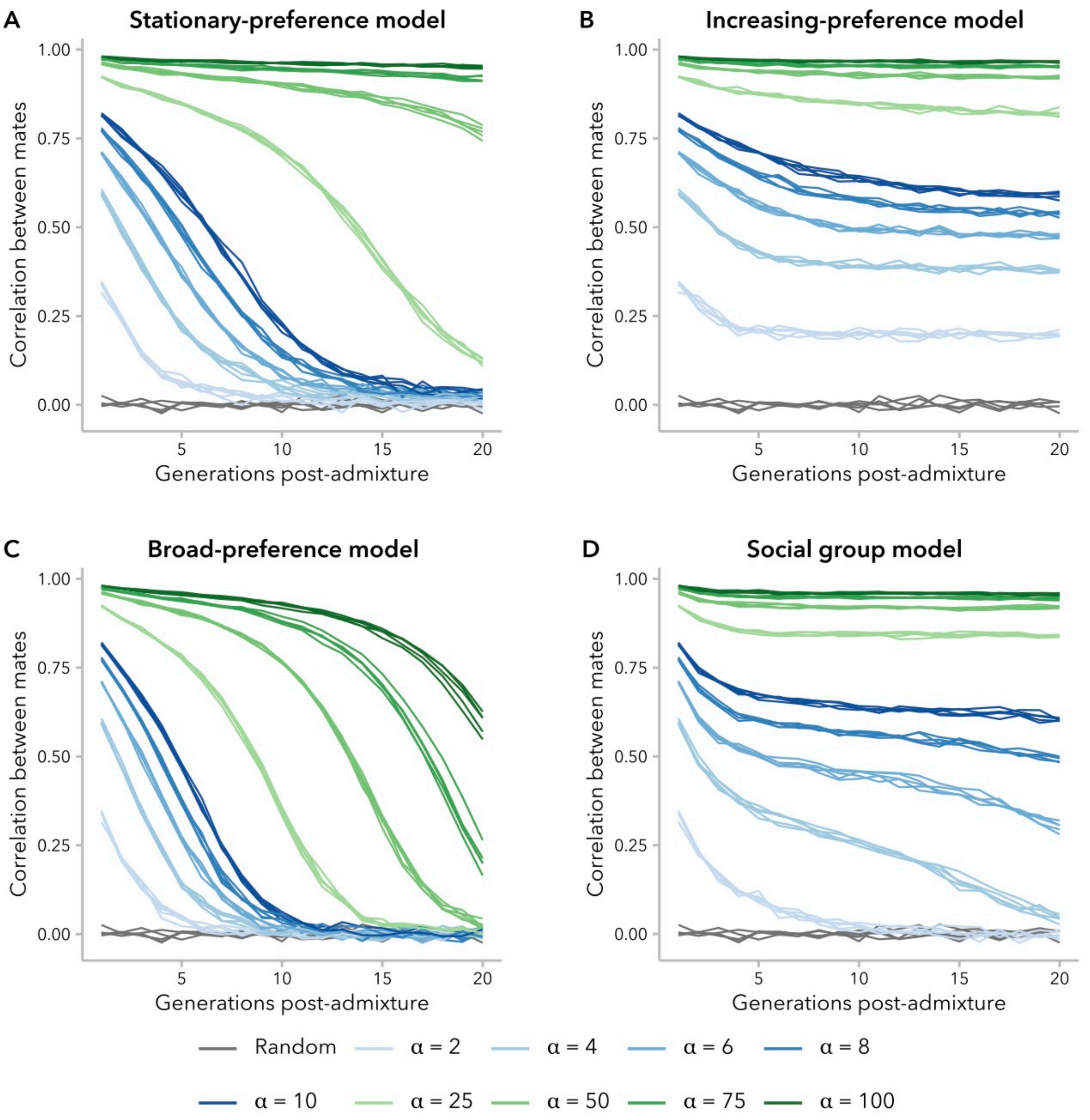

Figure S6. Correlation in ancestry between mates was not constant over time and decayed to near-zero in some simulations. For each model, higher values of α produced greater correlation in ancestry between mates in the early generations. In simulations under the stationary-preference and broad-preference models, the correlation in ancestry between mates was near-zero 20 generations post-admixture for $\alpha \leq 10$. The same random-mating simulations ($\alpha = 1$; gray) are reproduced in each subplot for reference. Compare to **Figure 2**.

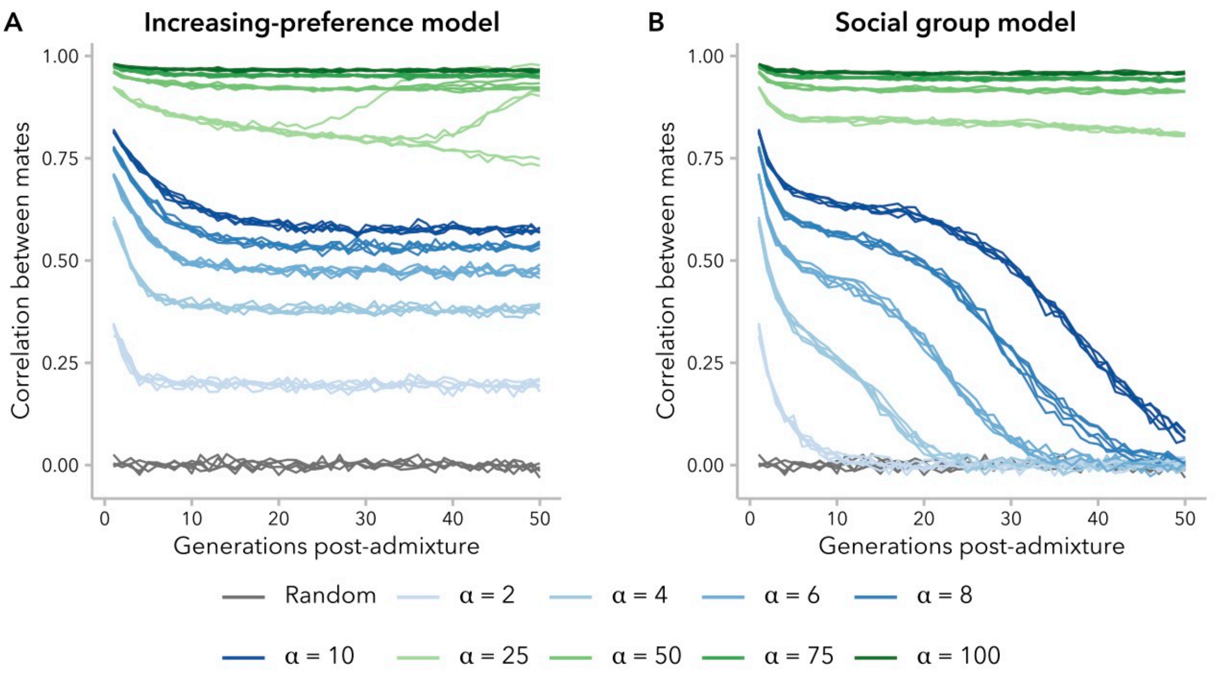

Figure S7. On longer time scales, the decay in correlation between mates differed between the increasing-preference and social groups models. **(A)** Correlation between mates reached a stable non-zero plateau maintained over 50 generations post-admixture under the increasing-preference model, for all α. **(B)** While correlations between mates were maintained for the first 20 generations post-admixture under the social group model (**Figure 2**), they decayed to zero on longer time scales.

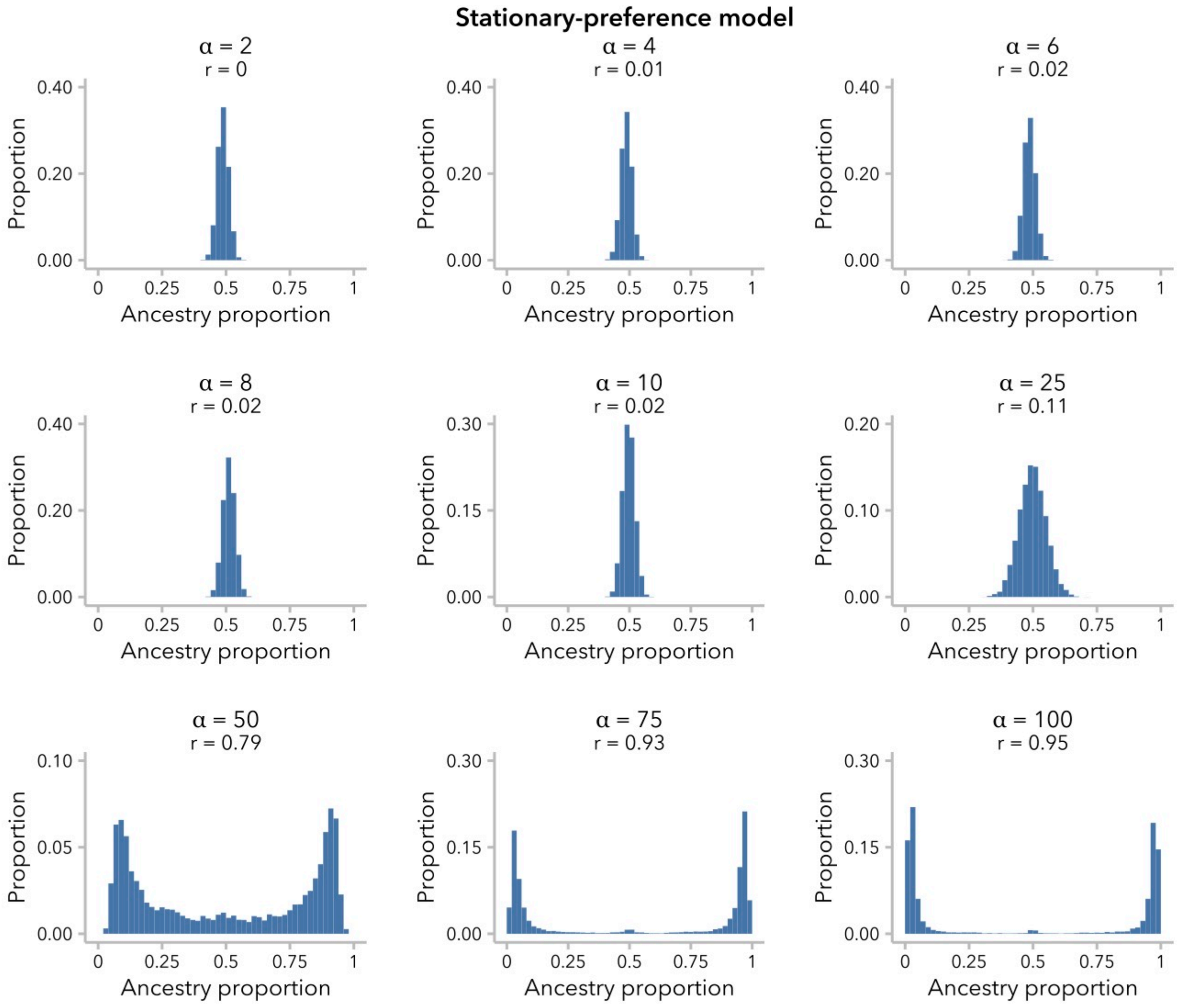

Figure S8. Under the stationary-preference model, a correlation in ancestry between mates was only observed at generation 20 when mate-choice bias was strong enough to largely prevent admixture ($\alpha > 10$). Each subplot displays the distribution of ancestry proportion at generation 20 for the indicated value of α.

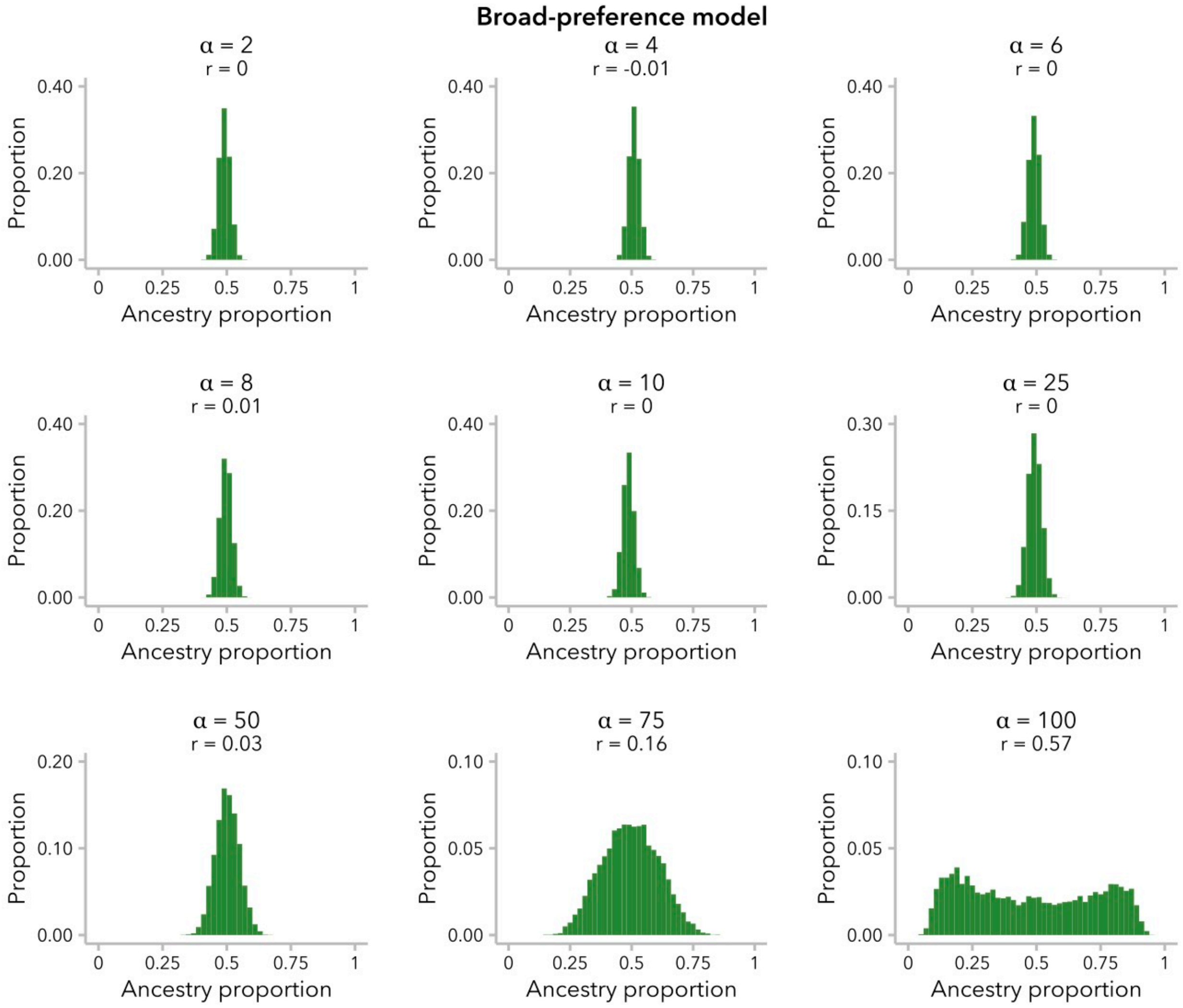

Figure S9. Under the broad-preference model, a correlation in ancestry between mates was only observed at generation 20 when mate-choice bias was strong enough to largely prevent admixture ($\alpha > 10$). Each subplot displays the distribution of ancestry proportion at generation 20 for the indicated value of α.

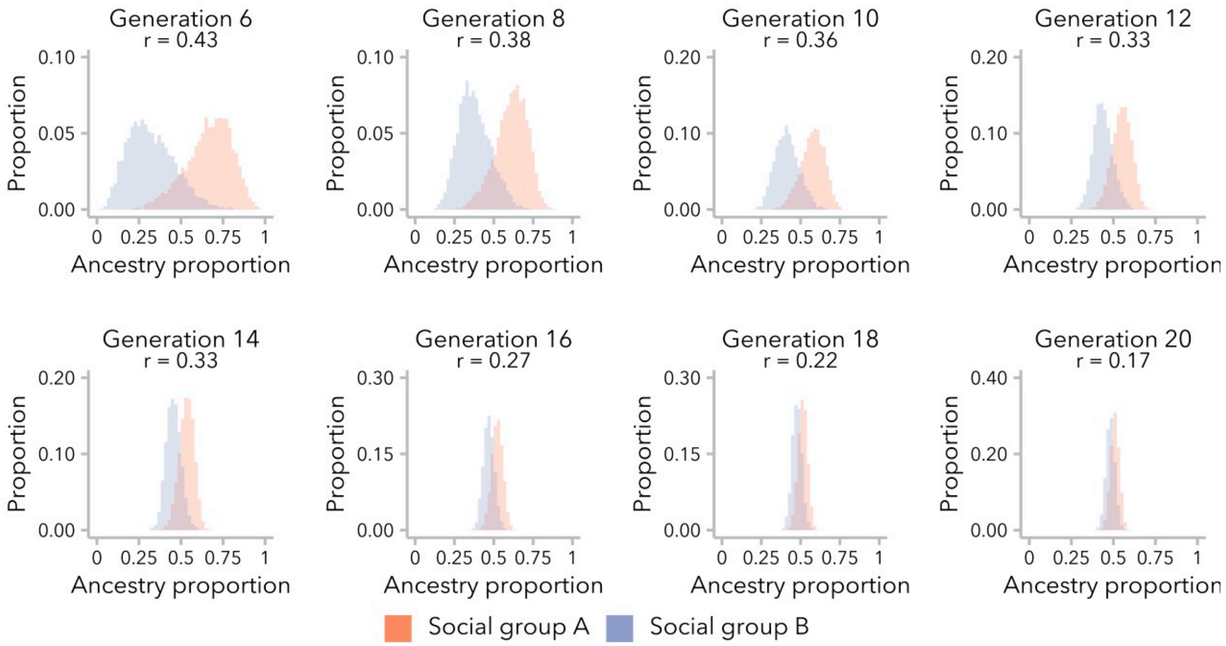

Figure S10. Differences in ancestry proportion between social groups became smaller over time, in simulations under the social group model. Despite increasing overlap in the distributions, correlation in ancestry between mates persisted 20 generations post-admixture. A representative example simulation is shown for $\alpha = 5$.

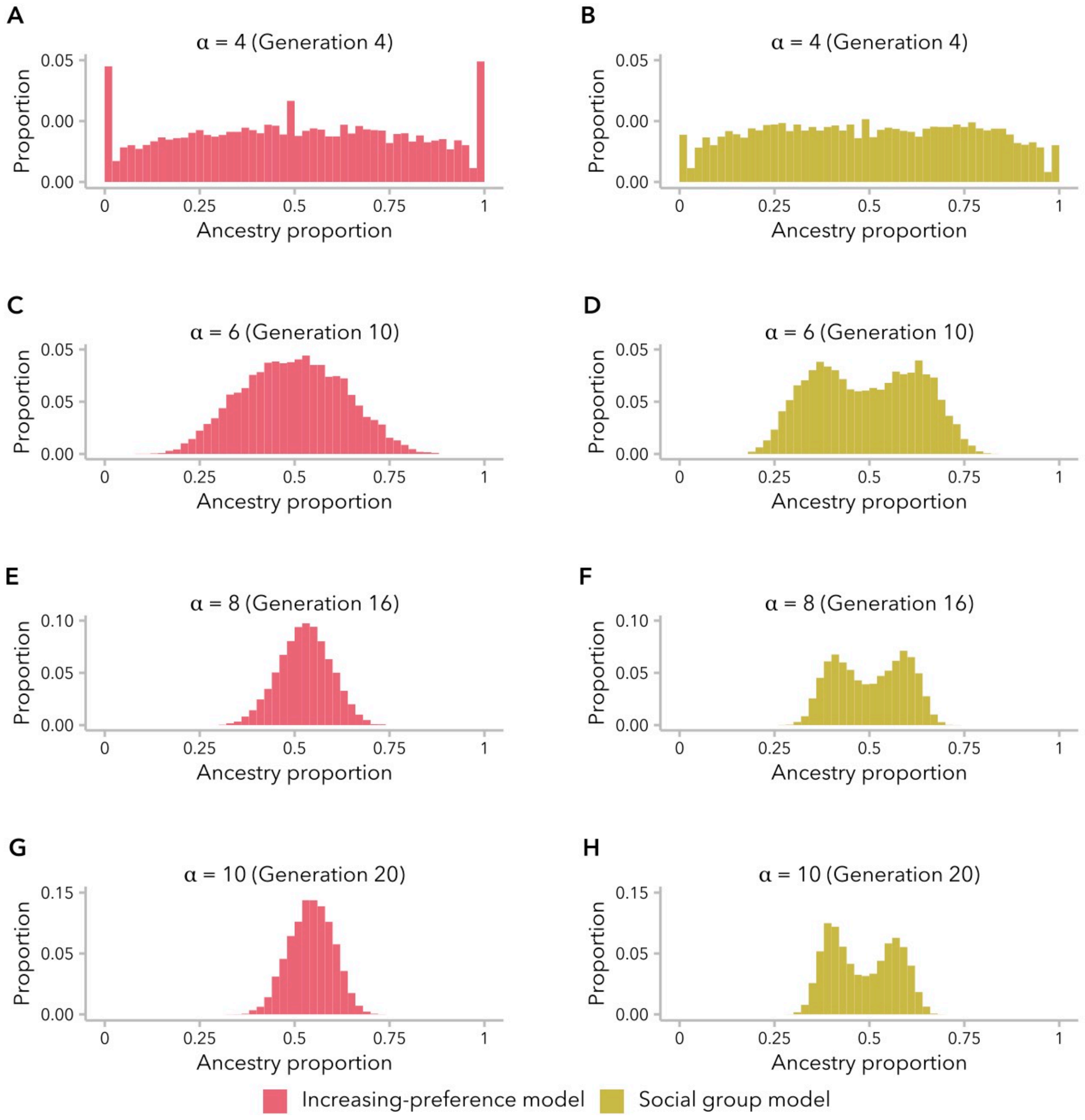

Figure S11. The transition from bimodal distribution of ancestry proportion to unimodal distribution was delayed in the social group model **(B, D, F, H)** relative to the increasing-preference model **(A, C, E, G)** for the same generation and α. Four representative examples are shown.

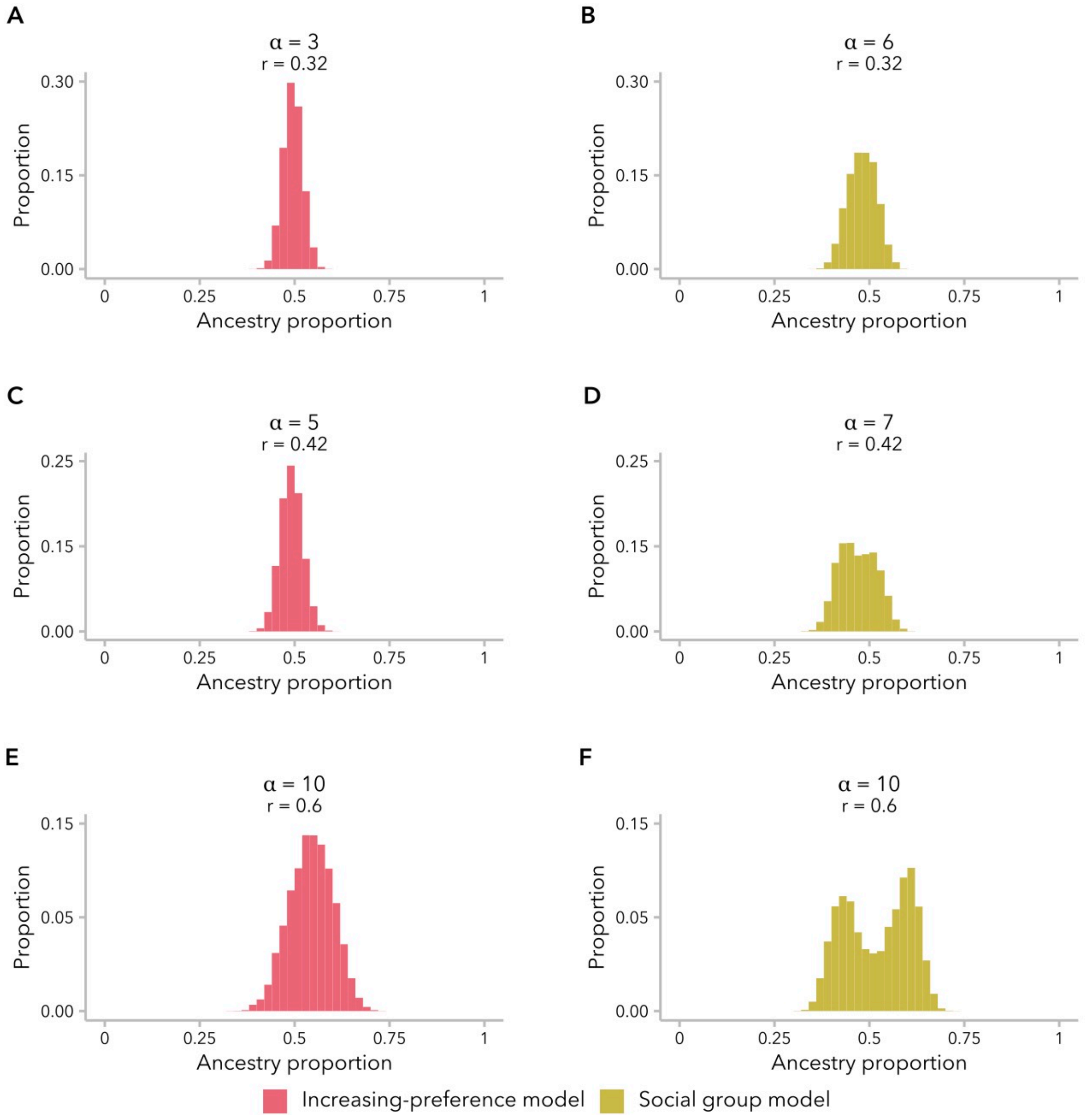

Figure S12. The distribution of ancestry proportion was unimodal 20 generations post-admixture in simulations under the increasing-preference model **(A, C, E)** and remained bimodal in simulations under the social group model **(B, D, F)**. The bimodality of the distribution was more pronounced for larger values of α. Three examples are shown, comparing simulations with similar correlation in ancestry between mates at generation 20.

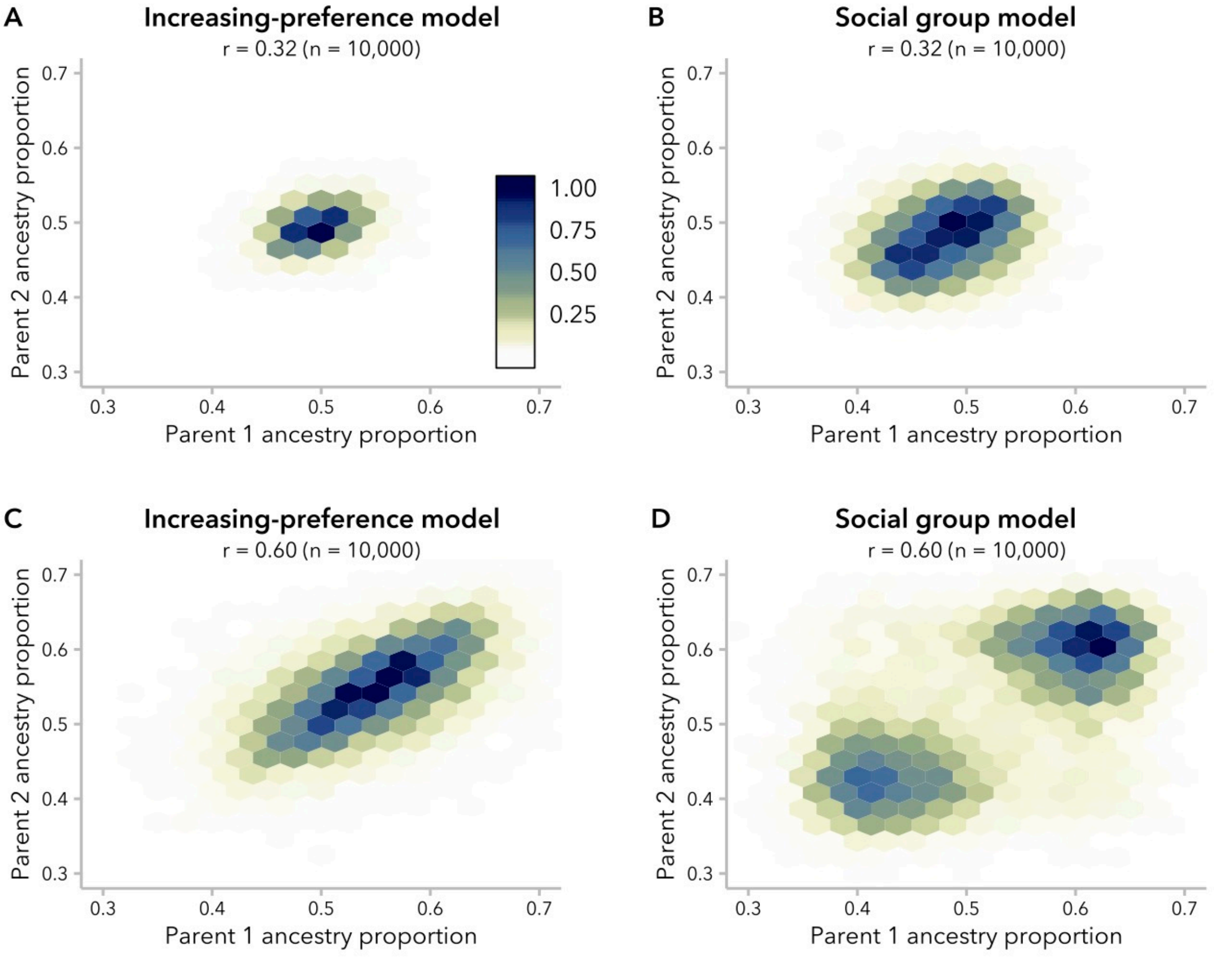

Figure S13. Differences in mating structure between simulations under the increasing-preference **(A, C)** and social group **(B, D)** models were more pronounced when the correlation in ancestry between mates was larger. All individuals in generation 20 are shown. Each hexagon represents a bin of 0.025 ancestry proportion units, colored by density scaled to a maximum value of 1. **(A)** $\alpha = 3$ **(B)** $\alpha = 6$. **(C-D)** $\alpha = 10$. Compare to **Figure 3A.**

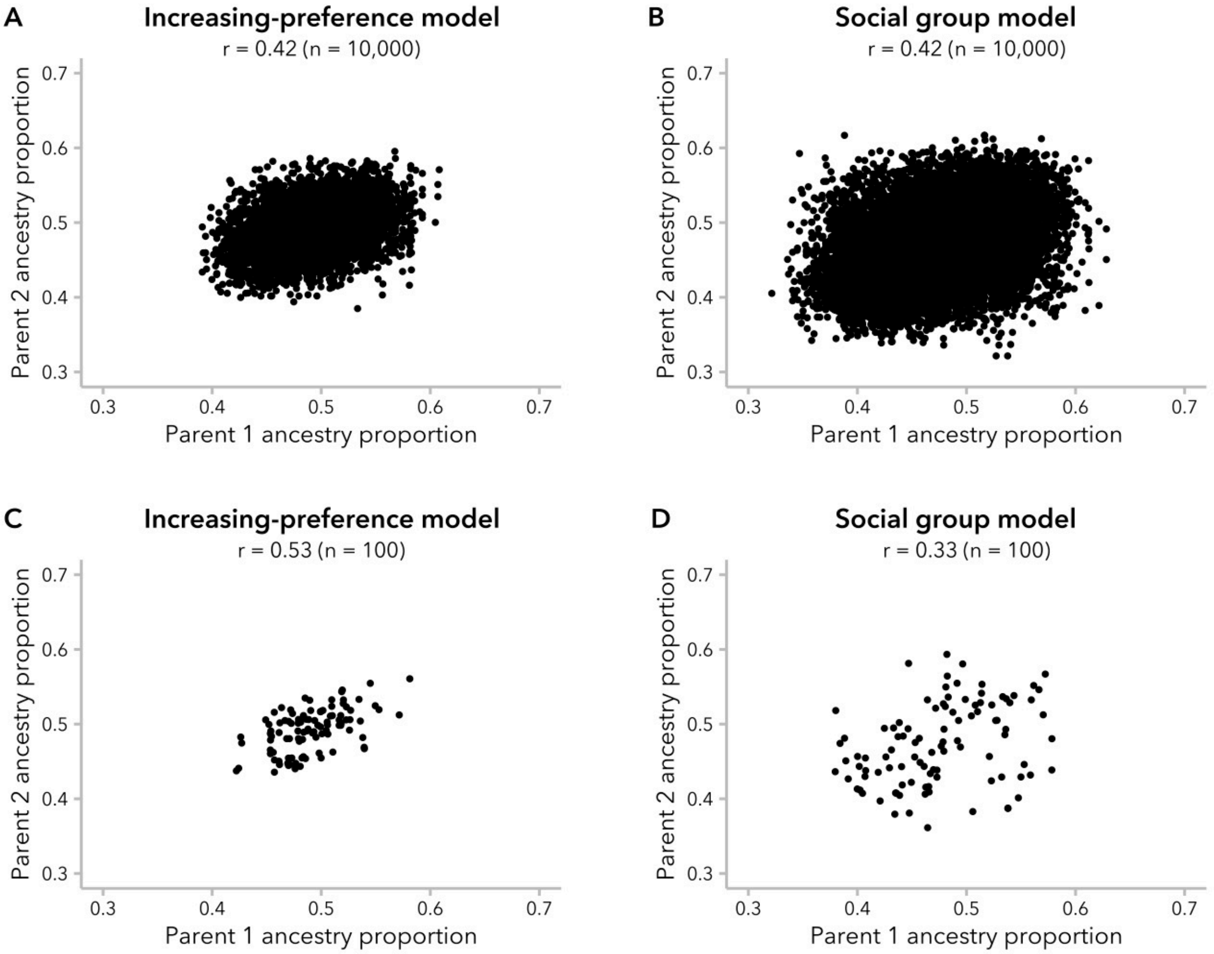

Figure S14. Simulations under the increasing-preference ($\alpha = 5$) and social group ($\alpha = 7$) models produced similar correlation coefficients with different underlying mating structure. Differences in the underlying structure were difficult to ascertain when visualized as a dot-plot. **(A)** Correlation in ancestry proportion between the two parents of all individuals in generation 20. **(B)** Correlation in ancestry proportion between the two parents of a subset of 100 individuals in generation 20.

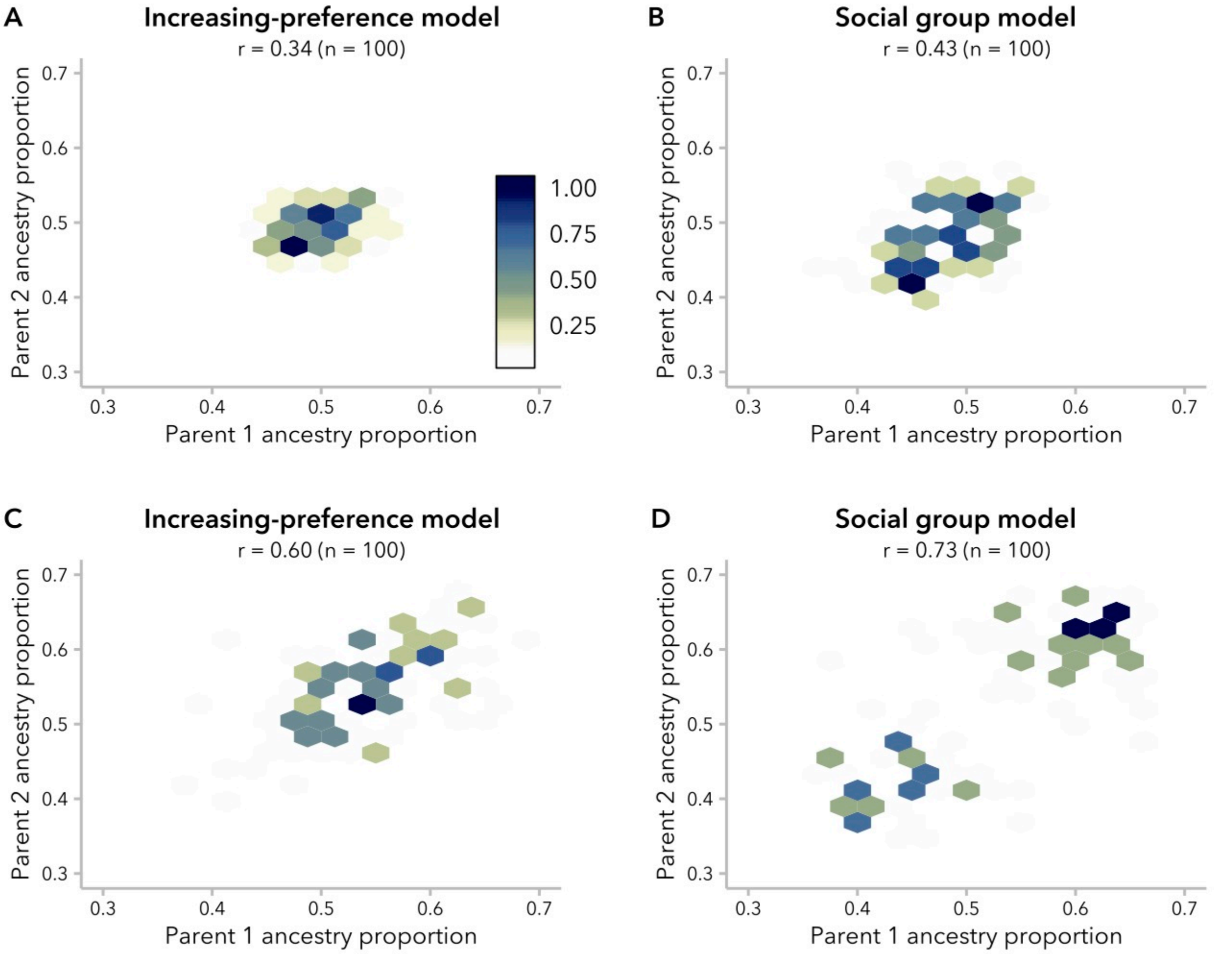

Figure S15. Even with a relatively small sample size ($n = 100$), "hexbin" plots can suggest differences between the increasing-preference (**A, C)** and social group **(B, D)** models. Differences in the structure of mating pairs is more apparent when the correlation in ancestry between mates is higher (**C, D**) . **(A)** $\alpha = 3$ **(B)** $\alpha = 6$. **(C-D)** $\alpha = 10$. Compare to **Figure 3C**.

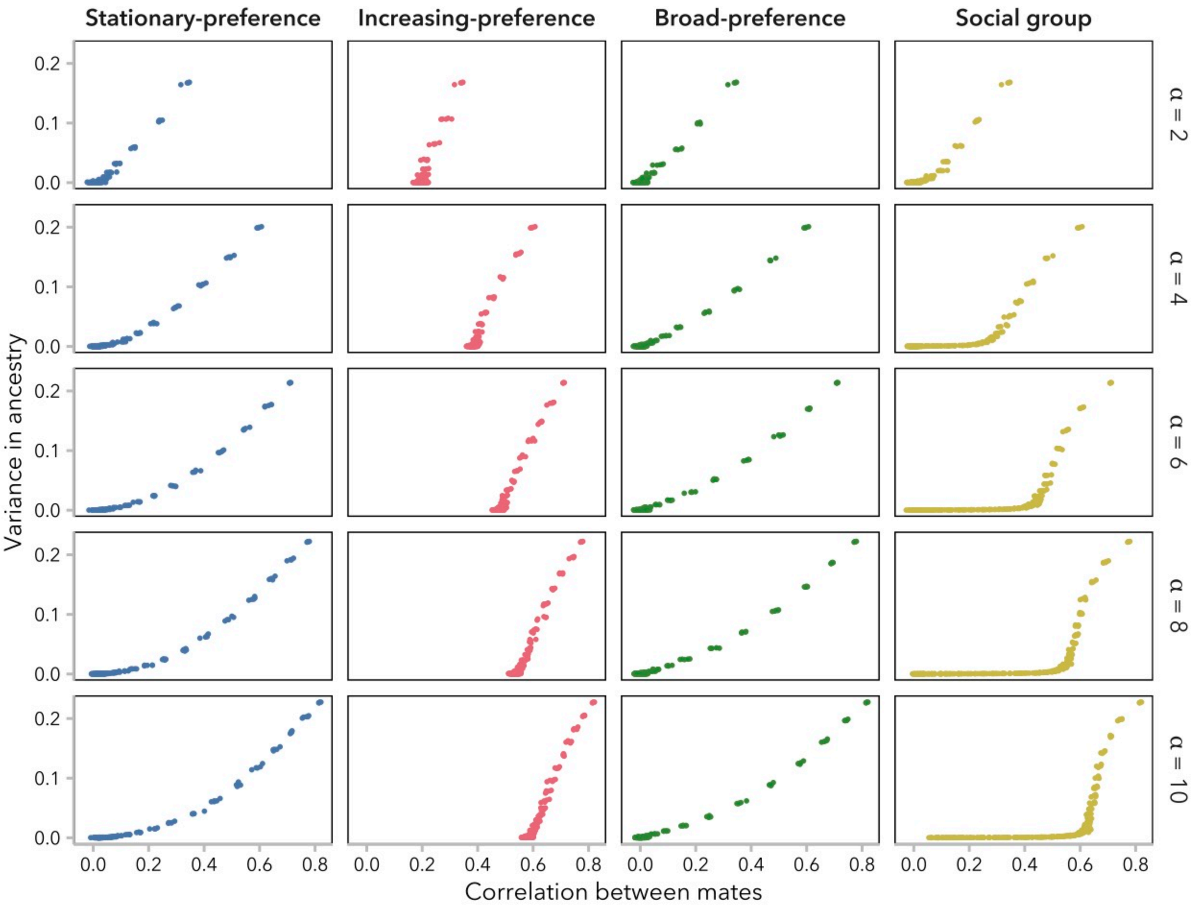

Figure S16. Correlation in ancestry proportion between mates is positively correlated with variance in ancestry proportion within the population across models and values of α. Relative to the other two models, the increasing-preference and social group models maintain a positive correlation even as variance decreases. Each dot represents one generation.

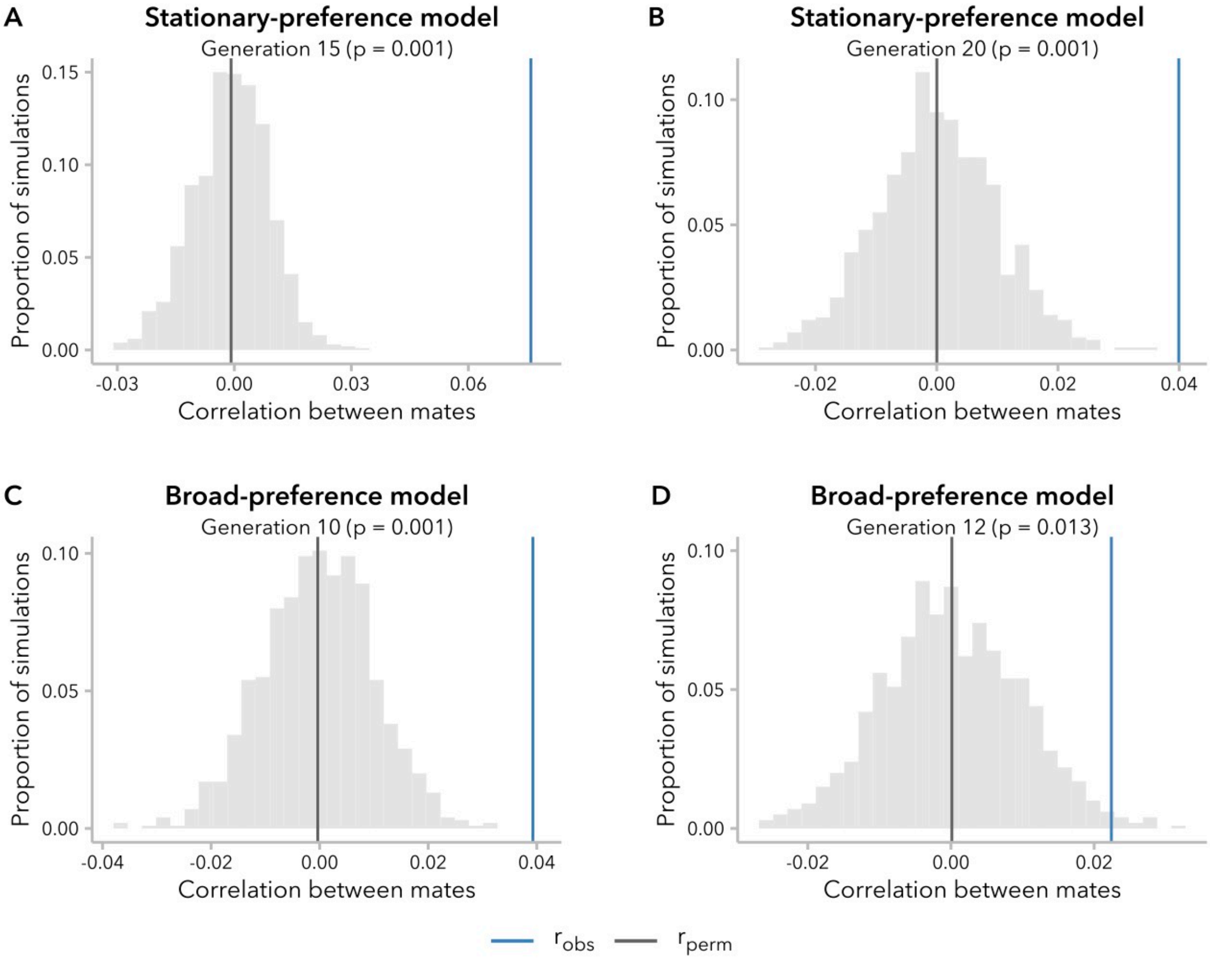

Figure S17. Mating can differ significantly from random even when the correlation in ancestry between mates is very small. **(A, B)** In simulations under the stationary-preference model, the correlation in ancestry between mates was significantly different from 1,000 permutations across all time points for $\alpha = 10$. **(C, D)** In simulations under the broad-preference model, the correlation between was only significantly different from permutation until generation 10 for $\alpha = 10$.

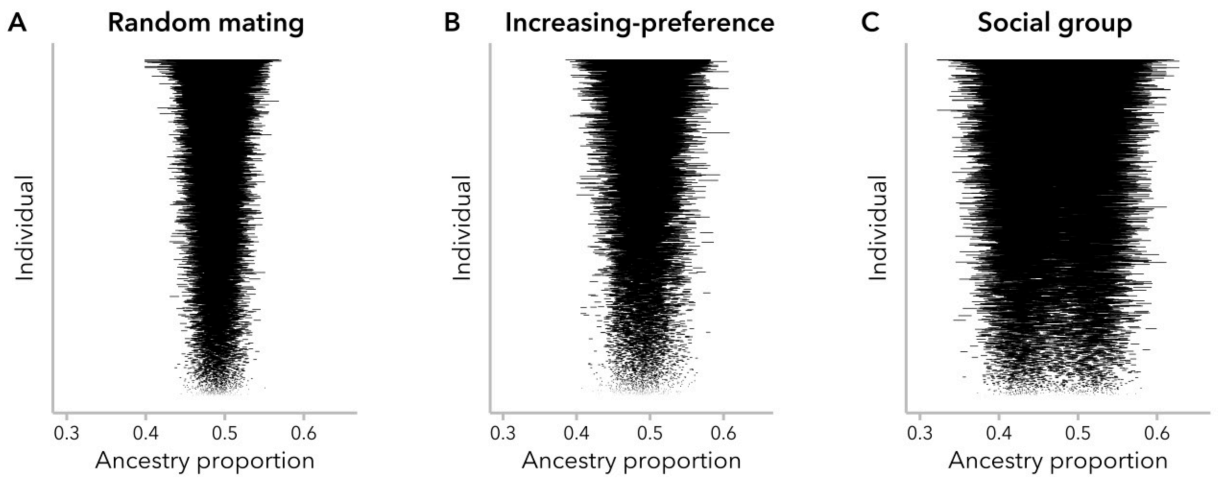

Figure S18. The difference in ancestry between mates, Δ_x, is an alternative to Pearson correlation coefficient for measuring the degree to which mating is biased. Representative examples are shown for **(A)** random mating ($\alpha = 1$), **(B)** the increasing-preference model ($\alpha = 10$), and **(C)** the social group model ($\alpha = 10$). Each line represents one individual in generation 20, connecting the ancestry proportion of parent 1 and parent 2.

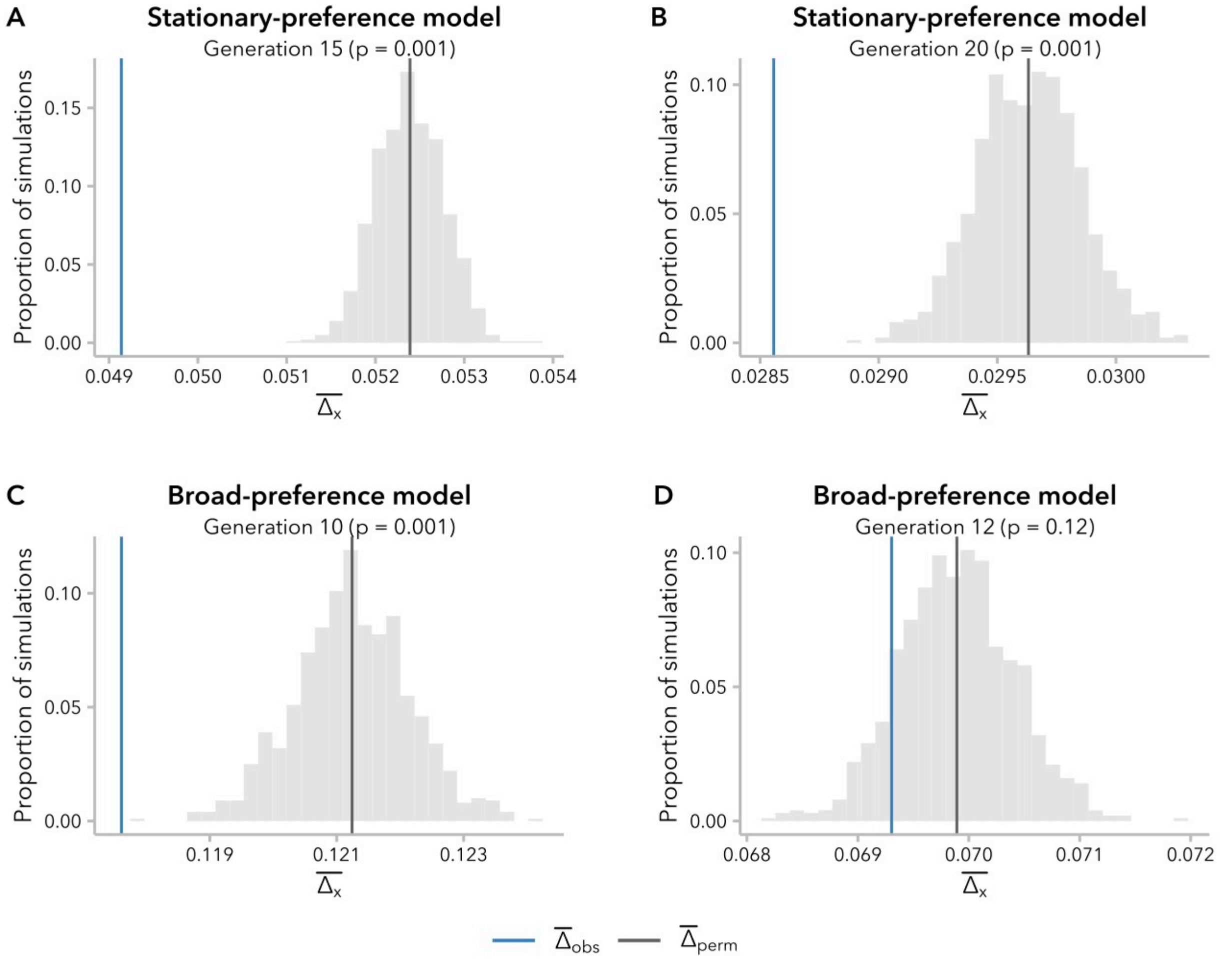

Figure S19. Comparison of Δ_x to permutations indicates that mating does not always differ from random, even when mate-choice is biased. **(A, B)** In simulations under the stationary-preference model, the mean difference in ancestry between mates, $\bar{\Delta}_{obs}$, was significantly different from the distribution of $\bar{\Delta}_{perm}$ for 1,000 permutations across all time points for $\alpha = 10$. **(B)** In simulations under the broad-preference model, $\bar{\Delta}_{obs}$ was only significantly different from the distribution of $\bar{\Delta}_{perm}$ for 1,000 permutations until around generation 10 for $\alpha = 10$.

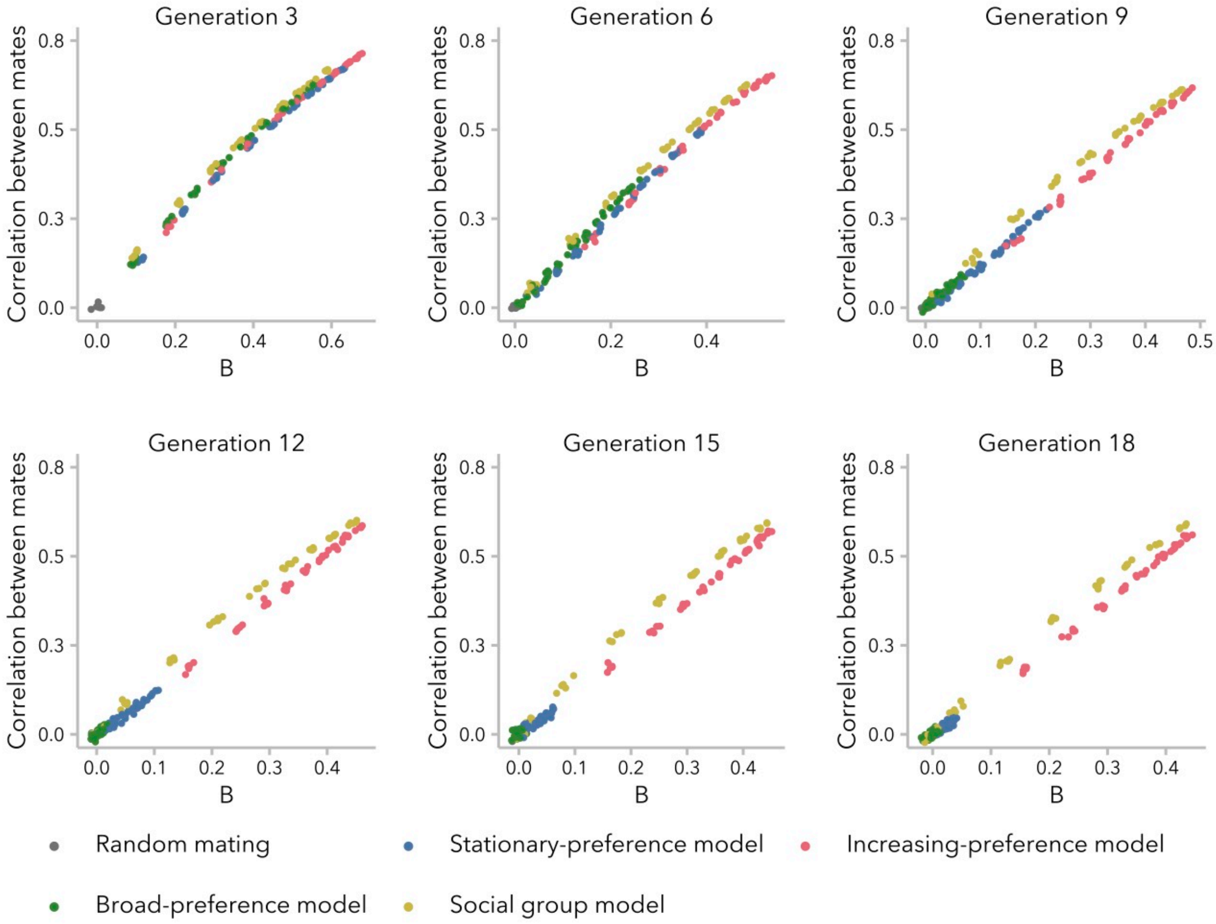

Figure S20. The relationship between the correlation coefficient for ancestry between mates and expressed mating bias B was similar across models and time points. Each dot represents one simulation. Compare to **Figure 5A.**

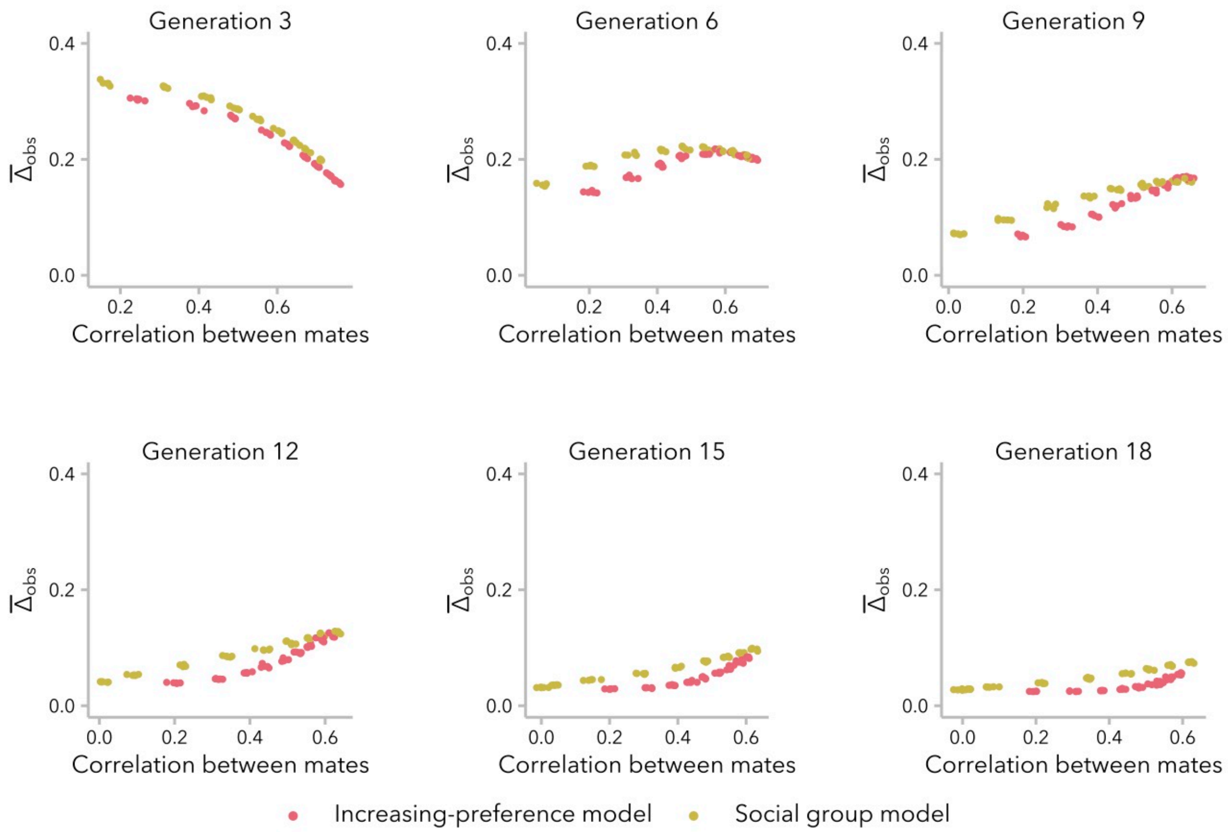

Figure S21. Direct comparison of $\bar{\Delta}_{obs}$ between models (instead of the variance-standardized B) demonstrates that for a given correlation in ancestry between mates, individuals are less similar to their mates under the social group model than under the increasing-preference model. The relationship between these variables is not consistent between generations. Each dot represents one simulation with 5 replicates per α.

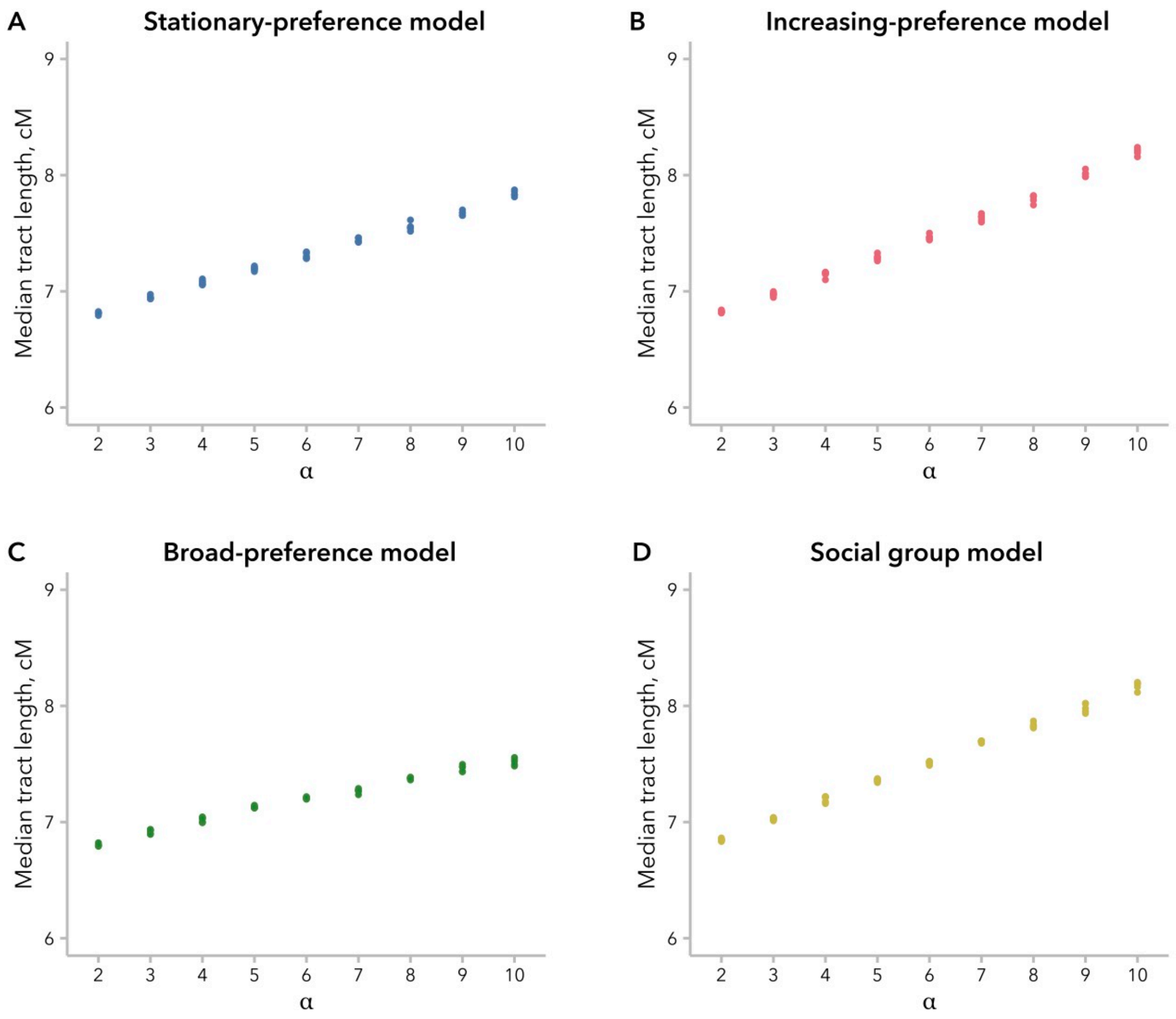

Figure S22. Comparing within each model, larger values of α consistently correspond to longer median local-ancestry tract lengths. Five replicate simulations are shown for each value of α.

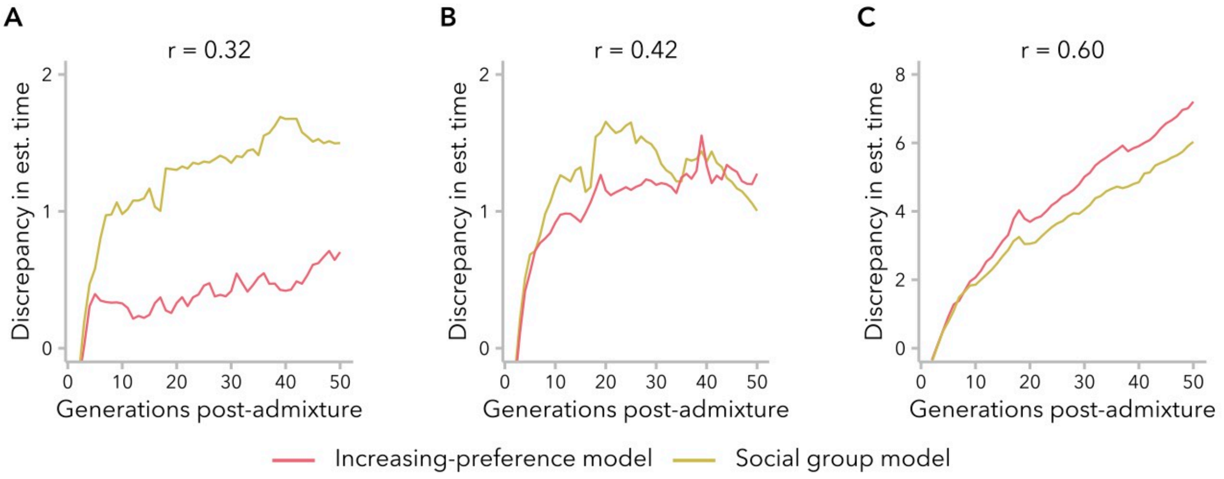

Figure S23. Underestimation of the time since admixture is more strongly influenced by earlier generations. In simulations under either the increasing-preference or social group model, the discrepancy between the estimated time since admixture and the true time since admixture did not increase linearly over time, and in some cases appeared to reach a plateau. Each line represents a single simulation.

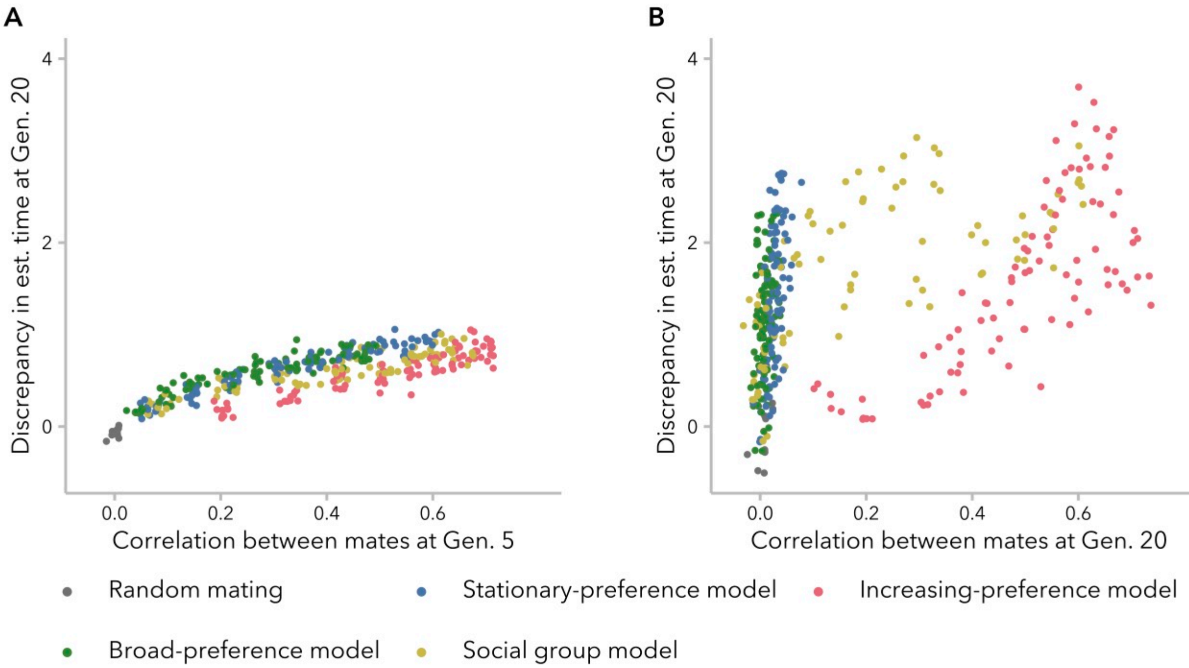

Figure S24. Underestimation of the time since admixture is more strongly influenced by earlier generations Correlation in ancestry between mates at generation 5 more strongly influenced the discrepancy in estimated time since admixture observed at generation 20 ($r = 0.89$) than did the correlation in ancestry between mates at generation 20 ($r = 0.46$).

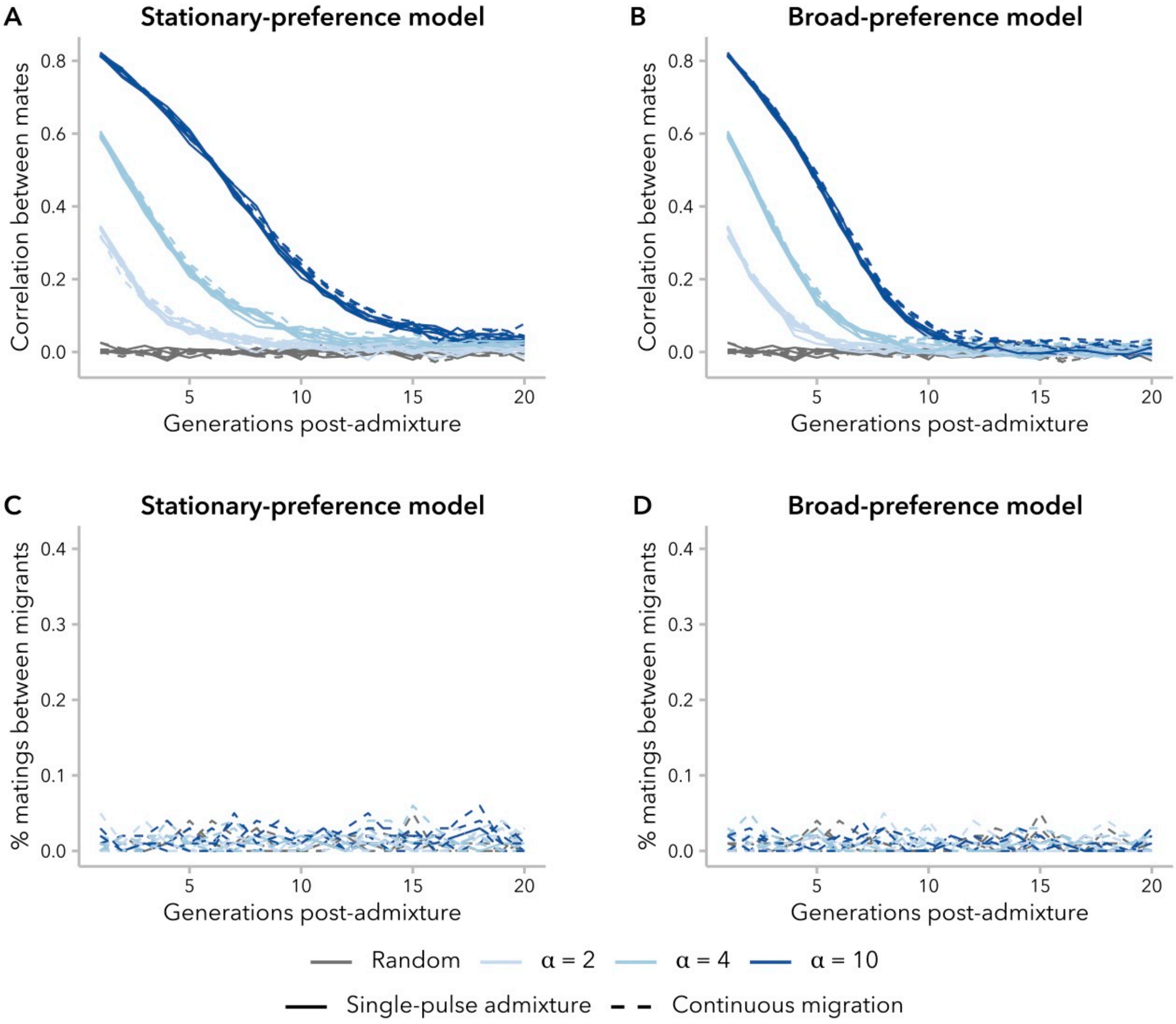

Figure S25. The effects of continuous migration differed between mate-choice models. **(A, B)** Correlation in ancestry between mates did not differ between simulations with and without migration, for the stationary-preference and broad preference models. **(C, D)** Under both models with continuous migration, mating between migrants did not occur more often than expected by chance.

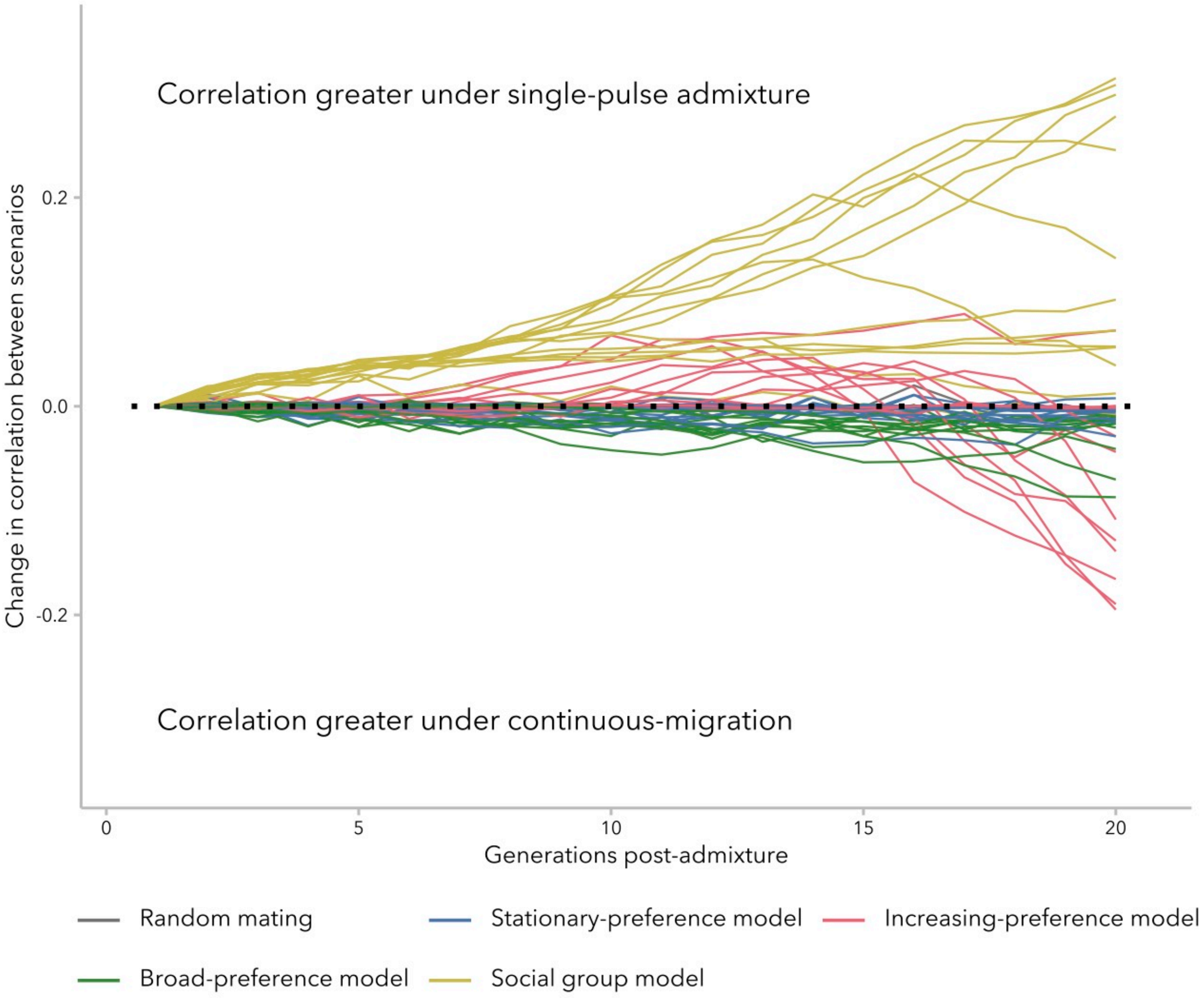

Figure S26. The effects of continuous migration differed between mate-choice models. The introduction of continuous migration tended to increase the correlation between mates under the increasing-preference model, relative to a scenario of pulse-admixture. The opposite trend was observed under the social group model. Correlation coefficients were generally unaffected by migration under the stationary-preference and broad-preference models.

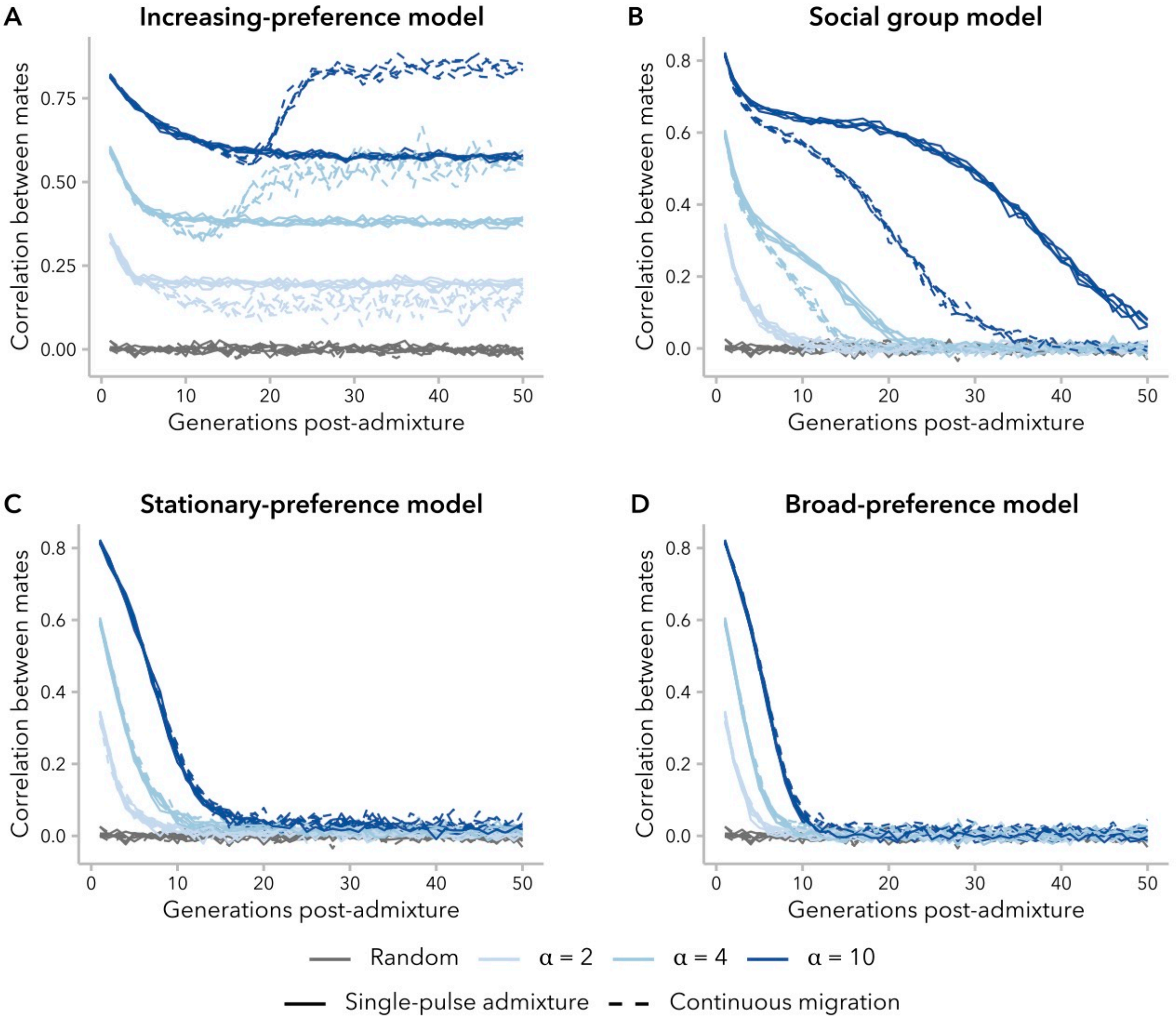

Figure S27. The disparate impacts of continuous migration between models were consistent on longer time scales. Correlation between mates with and without continuous migration is shown under each model for 50 generations post-admixture.
